# Characterizing the Distribution of *Oncorhynchus mykiss* Genetic Diversity in the Klamath River Basin Before Dam Removal

**DOI:** 10.1111/eva.70297

**Published:** 2026-07-12

**Authors:** Kathleen G. O'Malley, Mark E. Hereford, Kevin C. Olsen, Devon E. Pearse, William R. Tinniswood, Ben S. Ramirez, Jonathan B. Armstrong, Stanley J. Piotrowski

**Affiliations:** ^1^ State Fisheries Genomics Lab, Coastal Oregon Marine Experiment Station, Department of Fisheries, Wildlife, and Conservation Sciences, Hatfield Marine Science Center Oregon State University Newport Oregon USA; ^2^ Klamath Watershed District Office Oregon Department of Fish and Wildlife Klamath Falls Oregon USA; ^3^ Southwest Fisheries Science Center National Oceanic and Atmospheric Administration Santa Cruz California USA; ^4^ Department of Ecology and Evolutionary Biology University of California Santa Cruz California USA; ^5^ Department of Fisheries, Wildlife, and Conservation Sciences Oregon State University Corvallis Oregon USA

**Keywords:** connectivity, dam removal, genetic diversity, Klamath, *O. mykiss*, redband trout, steelhead

## Abstract

Genetic assessments serve as powerful tools to evaluate the effects of anthropogenic habitat fragmentation on natural populations. In the Klamath River Basin, construction of four dams from 1912 to 1962 had a profound impact on the distribution of anadromous fishes, blocking access to over 751 river kilometers of critical habitat. To characterize the genetic diversity and connectivity among *Oncorhynchus mykiss* in the basin prior to the historic removal of the four dams in 2024, we genotyped 2466 samples at 193 presumably neutral and 105 putatively adaptive markers. Using complementary population genetic analyses, we found a clear division between coastal and inland *O. mykiss* lineages located primarily downstream and upstream of the outlet of Upper Klamath Lake, and little genetic structure associated with dams. Further, we detected distinct inland and coastal genetic lineages upstream of the lake outlet supporting the hypothesis that ancestral inland *O. mykiss* were secondarily invaded by a coastal lineage. We found that neutral genetic diversity was significantly greater in collections consisting primarily of anadromous *O. mykiss* compared to collections of adfluvial, fluvial, and resident fish while genetic diversity was significantly lower in adfluvial collections. Based on the chromosome *Omy5* markers associated with anadromous/resident phenotypes, we found that anadromous and heterozygous genotypes were more prevalent downstream of the lake outlet while resident genotypes were prevalent upstream of the outlet. Based on the chromosome *Omy28* markers associated with adult migration timing, we found that late‐migration timing and heterozygous genotypes were numerous downstream of the lake outlet while early‐migration timing genotypes were prevalent upstream of the outlet. The results of our population genetic analyses highlight the genetic diversity and structure of *O. mykiss* in the Klamath River Basin and will serve as a critical baseline for future assessments post dam removal.

## 1 Introduction

Anthropogenic habitat fragmentation limits the dispersal of individuals, which may in turn reduce gene flow and lead to increased population structure (Benson et al. 2016; Schlaepfer et al. 2018; Winans et al. 2018). This decreased connectivity could result in higher rates of genetic drift and inbreeding and ultimately reduce evolutionary potential due to loss of genetic diversity (Frankham 2005; Pavlova et al. 2017). Furthermore, life history strategies may be disrupted if individuals are unable to access habitat required for feeding and breeding (McClure et al. 2008). This is particularly detrimental for freshwater fishes that move among habitats at different life stages and anadromous fishes that migrate between the ocean and freshwater environments. Liermann et al. (2012) reported that of the 397 freshwater ecoregions assessed globally, nearly 50% are obstructed by large‐ and medium‐size dams, and approximately 27% face additional downstream obstruction. This restricted connectivity will likely threaten biodiversity within freshwater ecosystems (Brauer and Beheregaray 2020).

**FIGURE 1:**
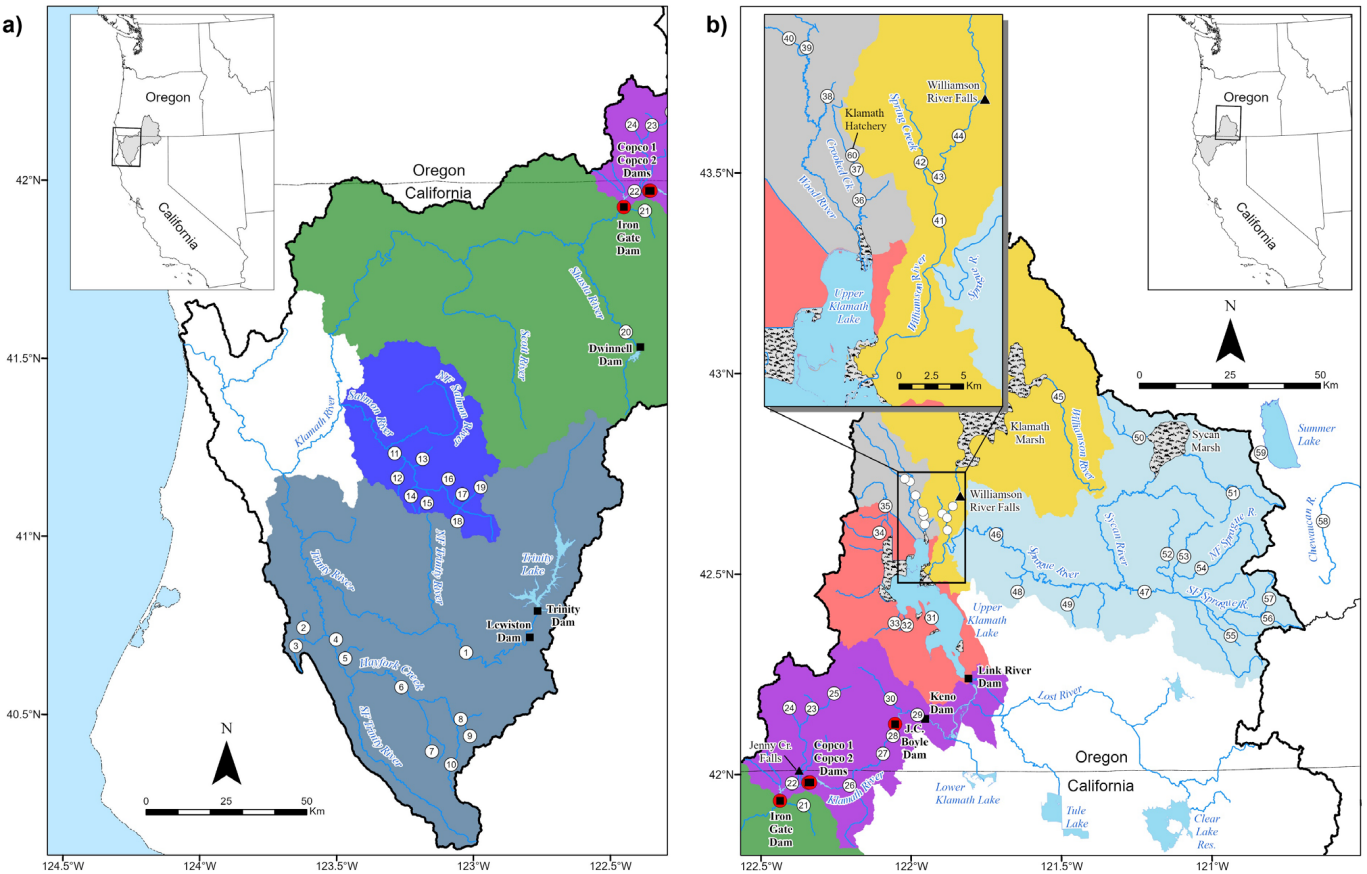
Maps of *O. mykiss* sampling collections in (a) the Lower Klamath River Basin and (b) the Upper Klamath River Basin of Southern Oregon and Northern California. Black squares indicate relevant dams and black triangles are waterfalls that block upstream passage. Dams removed after sample collection are indicated by black squares within red circles. Watersheds colors correspond to colors used in the ordinations.

The Klamath River Basin is a major watershed (40,631 km^2^) in southern Oregon and northern California, USA (Figure 1a,b) that has been impacted by anthropogenic habitat fragmentation since the early 20th century. The Klamath River is formed by headwater streams originating from both groundwater springs and precipitation sources that flow into Upper Klamath Lake (260 km^2^ surface area), a major natural feature that divides the upper and lower watersheds. The natural outlet of Upper Klamath Lake is regulated by Link River Dam (constructed in 1927) which maintains lake levels to support irrigation and sustains flows downstream to the Klamath River. Historically, this basin supported abundant runs of anadromous fishes from the headwaters in Oregon down to the Pacific Ocean (reviewed in Hamilton et al. 2005). However, construction of four hydroelectric dams below Upper Klamath Lake from 1912 to 1962 had a profound impact on the distribution of anadromous fishes in this basin, blocking access to over 751 river kilometers of spawning, incubation and rearing habitat for 112 years (Oregon Department of Fish and Wildlife and The Klamath Tribes 2021). Since then, the limit of upstream migration for anadromous fishes in the basin was Iron Gate Dam at river kilometer 312, which ultimately marked the division between the Lower Klamath River Basin and the Upper Klamath River Basin for 62 years.

The license to operate these four hydroelectric dams, referred to as the Klamath Hydroelectric Project (FERC Project No. 2082), expired in 2006. Since relicensing required establishing volitional passage of anadromous fishes as well as associated water quantity and quality measures, the 2010 Klamath Hydroelectric Settlement Agreement and associated 2016 amended version were developed to initiate removal of the four dams. This effort began in Fall 2023 and was completed a year later. To date, it represents the largest dam removal project in world history, opening the entire upper Klamath watershed to anadromous fish. The two remaining dams, Link River and Keno, are located at river kilometers 414 and 380, respectively, and have always contained fish passage facilities, though efforts are currently underway to improve passage specifically for anadromous fishes.

**FIGURE 2:**
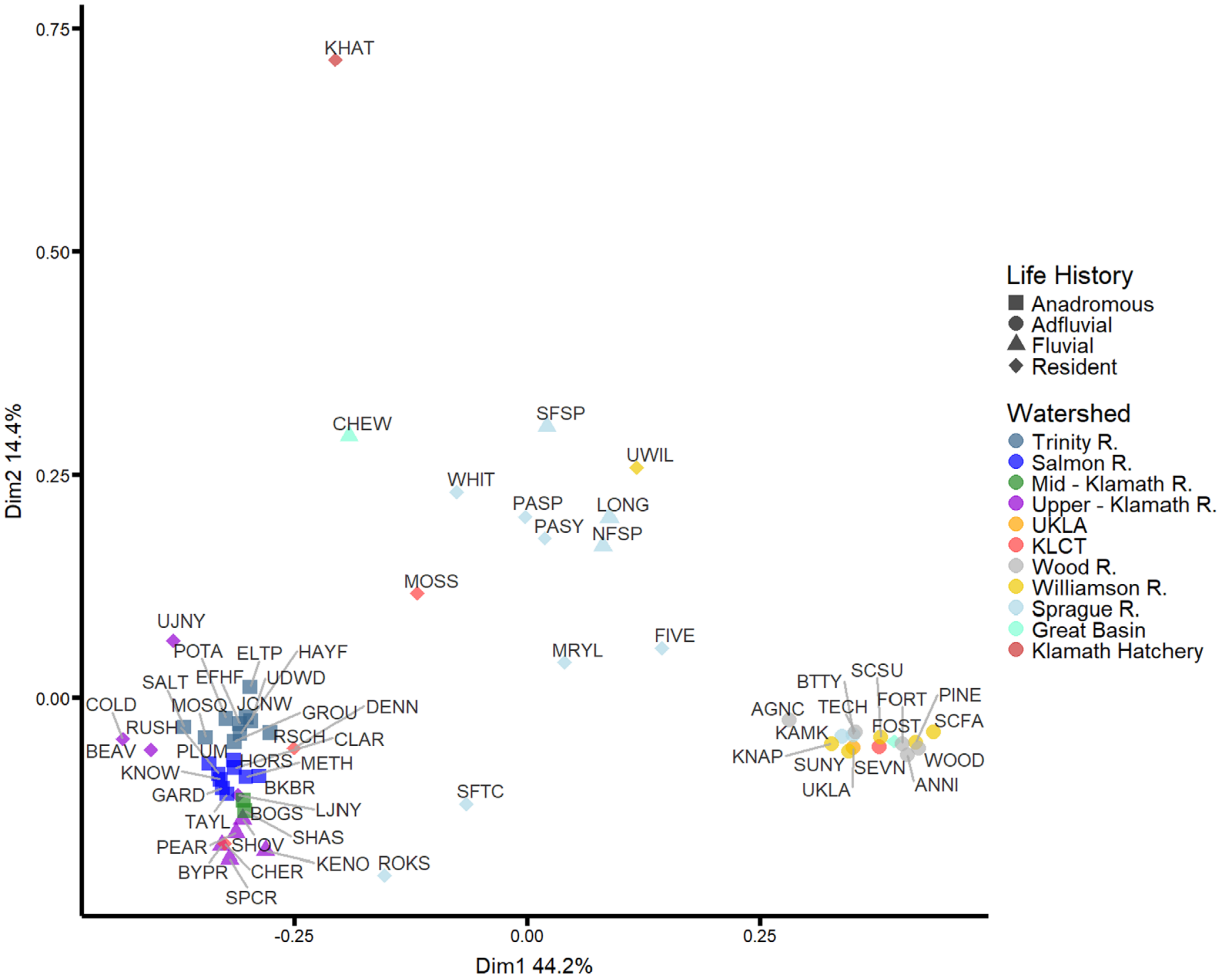
Population‐level ordination of *O. mykiss* contemporary collections (*n* = 2081 individuals from 61 collections within 11 watersheds) based on the neutral marker dataset. Shape and color of data points indicate the dominant life history type and watershed of each sampling collection, respectively.

The salmonid fish, *Oncorhynchus mykiss* , displays enormous life history variation including both ocean migrating (steelhead) and freshwater resident (rainbow trout) forms which can occur in sympatry (Behnke 2002). The Klamath River Basin broadly supports at least two lineages/subspecies of *O. mykiss* : (1) coastal rainbow trout and steelhead ( *O. mykiss irideus* ), which occupy the mainstem Klamath River and its tributaries below Upper Klamath Lake (Figures 1a and 2) inland Klamath redband trout (*O. mykiss newberii*), which inhabit diverse aquatic habitats in Upper Klamath Lake and its tributaries and co‐occur with coastal rainbow trout/steelhead trapped above the dams (Figure 1b) (Pearse et al. 2011). In the lower basin, 38 distinct *O. mykiss* life history categories at maturity have been identified which differ in duration of freshwater and ocean rearing, age at maturation, and incidence of repeat spawning (Hodge et al. 2016). In the upper basin, freshwater resident fish may remain in isolated headwater streams (e.g., Sprague River tributaries), including above barrier waterfalls (e.g., above Williamson River Falls), and in other tributaries of the Klamath River throughout their life cycle. Fluvial forms, which migrate between larger and smaller streams, were found in the Klamath River between the dams prior to dam removal and also persist in the Sprague River watershed. Not only do these forms occupy different habitats, they also differ in their phenotypes (Behnke 1992). Lastly, adfluvial forms feed in Upper Klamath Lake during the spring and fall and migrate to groundwater spring dominated tributaries to spawn. In the summer, adfluvial forms seek cooler water temperature in spring dominated tributaries (Hahlbeck et al. 2021). It is important to note that non‐native rainbow trout stocks have historically been released throughout the Upper Klamath River Basin (Oregon Department of Fish and Wildlife unpublished records) and the extent of their genetic impact on naturally spawning native *O. mykiss* populations is not known.

Genomic studies of *O. mykiss* have identified two distinct chromosomal regions strongly associated with migratory life history variation. First, a large chromosomal inversion (Omy5), characterized by strong linkage disequilibrium (Phillips et al. 2006; Pearse et al. 2014), is associated with multiple juvenile developmental traits (Nichols et al. 2008), as well as adult age at maturity (Beulke et al. 2023; Goetz et al. 2024), with strong divergence between anadromous and resident populations (Pearse et al. 2014; Pearse, Barson, et al. 2019a). The inversion complex contains more than 1000 genes, including those associated with the process of smoltification, the complex physiological and morphological changes associated with the transition from life in freshwater to saltwater (Nichols et al. 2008; Pearse, Barson, et al. 2019a). While individual migratory tendency is influenced by genotype, sex, and growth rate (Pearse, Barson, et al. 2019a; Kelson et al. 2020; Rundio et al. 2021), surveying Omy5 variation across the species range can identify the distribution of standing genetic variation necessary for oceanic migration. For example, while most populations above waterfall barriers possess a high frequency of the resident‐association Omy5 variation, studies of central California *O. mykiss* populations located above dams with large reservoirs identified high frequencies of the anadromous haplotype, suggesting that these adfluvial populations have the potential to resume an anadromous life history if access to the ocean is restored (Pearse et al. 2014; Leitwein et al. 2017; Pearse and Campbell 2018; Abadía‐Cardoso et al. 2019).

Second, a region of chromosome 28 (Omy28), centered between the *greb1l* and r*ock1* genes, is strongly associated with the timing of migration from the ocean to freshwater habitats for breeding (Hess et al. 2016; Prince et al. 2017; reviewed by Waples et al. 2022). While *O. mykiss* life‐history phenotypes are complex, variation within some rivers can be characterized by two distinct adult migratory types (reviewed by Waples et al. 2022). Summer‐returning steelhead, referred to as “early migrators,” begin their migration in a sexually immature state, and mature while holding in freshwater habitats. In contrast, winter‐returning steelhead, referred to as “late migrators,” arrive in freshwater with more developed gonads and rapidly ascend rivers shortly before spawning. While the exact causal genetic variants within this region remain unknown, the peak of statistical association with adult migration timing spans *greb1l* and the intergenic region between the two genes suggesting that the causal variant might be regulatory (Micheletti, Hess, et al. 2018a; Thompson et al. 2019, 2020).

The primary aim of this study was to generate a comprehensive genetic baseline for *O. mykiss* in the Klamath River Basin as it existed prior to the largest dam removal project in world history. Using tissue samples collected prior to 2020 and a previously developed single nucleotide polymorphism (SNP) panel we had three primary objectives: (1) characterize the genetic diversity and connectivity among *O. mykiss* sampled from 74 collections in 11 watersheds of the Lower and Upper Klamath River Basin and the Great Basin (2) examine differences in genotype frequencies across collections at specific markers in the Omy5 and Omy28 regions associated with anadromy/residency and adult migration timing, respectively and (3) test for genetic differentiation in 13 locations with corresponding past (i.e., 2000–2006; Pearse et al. 2011) and contemporary (i.e., 2015–2019) collections. Given the recent dam removals and restoration of riverine connectivity throughout the Klamath River Basin, this genetic monitoring study will serve as a critical baseline for future monitoring efforts.

## 2 Methods

### 2.1 Sampling

In total, we evaluated 4280 *O. mykiss* tissue samples collected in 2000–2003, 2006, and 2015–2019 from 11 watersheds in the Lower and Upper Klamath River Basins and Great Basin of Southern Oregon and Northern California, USA (Figure 1, Tables 1, 2, Figure S1). Tissue samples were collected from juvenile (*n* = 2744), adult (*n* = 510), and individuals with undetermined life stages (*n* = 1026) by the Oregon Department of Fish and Wildlife (ODFW), California Department of Fish and Wildlife (CDFW), and University of California, Davis (U.C. Davis) using electrofishing, in‐stream weir, and hook‐and‐line methods. In addition, we included samples that were collected as part of Pearse et al. (2007) and Hahlbeck et al. (2023) to increase the breadth of sampling locations, and samples from Pearse et al. (2011) to opportunistically test for temporal genetic differentiation, primarily from 2000 to 2019 (Table 3) within a subset of collections. Collection date is included in Table S1.

**TABLE 1:** Summary of watersheds, location(s) and associated code, year(s) collected, sample sizes (*n*), life history type (LH), and expected heterozygosity (*H*_E_) estimated with the neutral marker dataset in collections downstream of Iron Gate Dam in the Lower Klamath River Basin. Life history types assessed included anadromous (A), adfluvial (ADF), fluvial (FLU), and resident (R).

#	Watershed	Location(s)	Code	Year(s) 20‐	*n*	LH	*H*_E_
1	Trinity R.	Junction City Weir	JCNW	15, 19	54	A	0.29
2	Trinity R.	Mosquito Cr.	MOSQ	16	14	A	0.28
3	Trinity R.	Grouse Cr.	GROU	16	13	A	0.27
4	Trinity R.	Underwood Cr.	UDWD	16	11	A	0.26
5	Trinity R.	Eltapom/Hyampom Cr.	ELTP	16	26	A	0.30
6	Trinity R.	Rusch Cr.	RSCH	16	12	A	0.28
7	Trinity R.	Salt/Philpot Cr.	SALT	16	15	A	0.27
8	Trinity R.	East Fork Hayfork Cr.	EFHF	16	12	A	0.29
9	Trinity R.	Potato Cr.	POTA	16	13	A	0.29
10	Trinity R.	Hayfork/Dubakella Cr.	HAYF	16	16	A	0.25
11	Salmon R.	Knownothing/McNeal Cr.	KNOW	16	16	A	0.27
12	Salmon R.	Methodist Cr.	METH	03	20	A	0.27
12	Salmon R.	Methodist/Indian Cr.	METH	16	21	A	0.27
13	Salmon R.	Black Bear Cr.	BKBR	16	20	A	0.27
14	Salmon R.	Plummer Cr.	PLUM	16, 17	15	A	0.28
15	Salmon R.	St. Claire Cr.	CLAR	16, 17	14	A	0.27
16	Salmon R.	Taylor/Shadow Cr.	TAYL	16	15	A	0.27
17	Salmon R.	Garden/Ray's Gulch	GARD	16, 17	24	A	0.27
18	Salmon R.	Blind Horse Cr.	HORS	16, 17	19	A	0.26
19	Salmon R.	Rush Cr.	RUSH	16, 17	20	A	0.27
20	Mid—Klamath R.	Shasta R.	SHAS	19	18	A	0.26
21	Mid—Klamath R.	Bogus Cr.	BOGS	19	17	A	0.27

**TABLE 2:** Summary of watersheds, location(s) and associated code, year(s) collected, sample sizes (*n*), life history type (LH), and expected heterozygosity (*H*_E_) estimated with the neutral marker dataset in collections upstream of Iron Gate Dam in the Upper Klamath River Basin and Great Basin. Life history types assessed included anadromous (A), adfluvial (ADF), fluvial (FLU), and resident (R). *UKLA consisted of multiple sampling locations in the Upper Klamath Lake; see Table S1 for details.

#	Watershed	Location(s)	Code	Year(s) 20‐	n	LH	*H*_E_
22	Upper—Klamath R.	Lower Jenny Cr.	LJNY	19	45	R	0.26
23	Upper—Klamath R.	Upper Jenny Cr.	UJNY	00	44	R	0.27
23	Upper—Klamath R.	Upper Jenny Cr.	UJNY	19	21	R	0.27
24	Upper—Klamath R.	Beaver Cr.	BEAV	19	38	R	0.20
25	Upper—Klamath R.	Cold Cr.	COLD	19	47	R	0.20
26	Upper—Klamath R.	Shovel Cr.	SHOV	19	28	FLU	0.25
27	Upper—Klamath R.	Peaking Reach	PEAR	18, 19	55	FLU	0.26
28	Upper—Klamath R.	J.C. Boyle/Bypass Reach	BYPR	00	38	FLU	0.25
28	Upper—Klamath R.	Bypass Reach	BYPR	19	28	FLU	0.25
29	Upper—Klamath R.	Keno Reach	KENO	00	12	FLU	0.24
29	Upper—Klamath R.	Keno Reach	KENO	16–19	78	FLU	0.24
30	Upper—Klamath R.	Spencer Cr.	SPCR	00	69	FLU	0.23
30	Upper—Klamath R.	Spencer Cr.	SPCR	17, 19	67	FLU	0.23
31	UKLA	Shoalwater/Howard Bay*	UKLA	16–18	27	ADF	0.14
32	KLCT	Denny Cr.	DENN	19	24	R	0.21
33	KLCT	Moss Cr.	MOSS	19	27	R	0.20
34	KLCT	Cherry Cr.	CHER	00	28	R	0.24
34	KLCT	Cherry Cr.	CHER	19	29	R	0.22
35	KLCT	Sevenmile Cr.	SEVN	18,19	54	ADF	0.13
36	Wood R.	Agency Cr.	AGNC	19	75	ADF	0.16
37	Wood R.	Tecumseh Cr.	TECH	19	44	ADF	0.14
38	Wood R.	Fort Cr.	FORT	19	78	ADF	0.11
39	Wood R.	Upper Wood R.	WOOD	19	75	ADF	0.10
40	Wood R.	Annie Cr.	ANNI	19	49	ADF	0.11
41	Williamson R.	Pine Ridge	PINE	19	101	ADF	0.11
42	Williamson R.	Spring Cr.	SCSU	00	15	ADF	0.12
42	Williamson R.	Spring Cr. Fall	SCFA	19	49	ADF	0.09
42	Williamson R.	Spring Cr. Summer	SCSU	19	77	ADF	0.13
43	Williamson R.	Sunnybrook Cr.	SUNY	17, 19	19	ADF	0.13
44	Williamson R.	Knapps Dam/Kirk	KNAP	17, 19	27	ADF	0.14
45	Williamson R.	Upper Williamson R.	UWIL	19	44	R	0.24
46	Sprague R.	Kamkaun Spring	KAMK	17, 19	10	ADF	0.14
47	Sprague R.	Beatty Gap/Spring Cr.	BTTY	17, 19	64	ADF	0.13
48	Sprague R.	South Fork Trout Cr.	SFTC	00	25	R	0.24
48	Sprague R.	South Fork Trout Cr.	SFTC	19	17	R	0.19
49	Sprague R.	Rock Cr.	ROKS	00	19	R	0.12
49	Sprague R.	Rock Cr.	ROKS	19	17	R	0.10
50	Sprague R.	Long Cr.	LONG	00	38	FLU	0.25
50	Sprague R.	Long Cr.	LONG	19	13	FLU	0.25
51	Sprague R.	Paradise Cr.	PASY	00	31	R	0.25
51	Sprague R.	Paradise Cr.	PASY	19	22	R	0.27
52	Sprague R.	Fivemile Cr.	FIVE	00	18	R	0.21
52	Sprague R.	Fivemile Cr.	FIVE	19	60	R	0.22
53	Sprague R.	Meryl Cr.	MRYL	19	28	R	0.25
54	Sprague R.	North Fork Sprague R.	NFSP	06	28	FLU	0.26
54	Sprague R.	North Fork Sprague R.	NFSP	19	41	FLU	0.26
55	Sprague R.	Paradise Cr.	PASP	19	25	R	0.19
56	Sprague R.	Whitworth Cr.	WHIT	18, 19	43	R	0.30
57	Sprague R.	South Fork Sprague R.	SFSP	19	21	FLU	0.27
58	Great Basin	Chewaucan R.	CHEW	19	38	FLU	0.26
59	Great Basin	Foster Cr.	FOST	17	13	R	0.07
60	Klamath Hatchery	Klamath Hatchery (53)	KHAT	19	78	R	0.28

**TABLE 3:** Temporal genetic differentiation in 13 locations with corresponding past and contemporary collections quantified with the neutral and adaptive marker datasets. Lower Klamath Basin (LKB) and Upper Klamath Basin (UKB), watershed, location code, year of past and contemporary collection(s), neutral and adaptive F_ST_ estimates with 95% confidence interval in parentheses are shown for each temporal comparison. Significant F_ST_ estimates in bold.

Basin	Watershed	Location Code	Year of 1st Collection	Year(s) of 2nd Collection	Neutral *F*_ST_	Adaptive *F*_ST_
LKB	Salmon R.	METH	2003	2016	**0.020 (0.010)**	**0.058 (0.038)**
UKB	Upper—Klamath R.	UJNY	2000	2019	**0.007 (0.006)**	0.010 (0.010)
UKB	Upper—Klamath R.	BYPR	2000	2019	−0.001 (0.003)	**0.012 (0.009)**
UKB	Upper—Klamath R.	KENO	2000	2016–2019	0.001 (0.006)	0.001 (0.007)
UKB	Upper—Klamath R.	SPCR	2000	2017, 2019	0.001 (0.002)	0.001 (0.004)
UKB	KLCT	CHER	2000	2019	**0.013 (0.007)**	**0.025 (0.014)**
UKB	Williamson R.	SCSU	2000	2019	0.006 (0.006)	0.010 (0.018)
UKB	Sprague R.	SFTC	2000	2019	**0.073 (0.021)**	**0.071 (0.029)**
UKB	Sprague R.	ROKS	2000	2019	**0.084 (0.034)**	**0.104 (0.060)**
UKB	Sprague R.	LONG	2000	2019	0.003 (0.006)	**0.018 (0.014)**
UKB	Sprague R.	PASY	2000	2019	**0.032 (0.011)**	**0.026 (0.015)**
UKB	Sprague R.	FIVE	2000	2019	0.002 (0.004)	0.001 (0.005)
UKB	Sprague R.	NFSP	2006	2019	**0.011 (0.007)**	0.007 (0.007)

We designated the most prevalent life history type (i.e., anadromous, adfluvial, fluvial, or resident) for each collection based on morphology, location, putative life stage (i.e., juvenile, adult, or unknown) and fork length (FL). The anadromous life history type occurs below Iron Gate Dam where steelhead can migrate between the ocean and freshwater environments and exists in sympatry with resident *O. mykiss* . Adfluvial, fluvial, and resident life history types occur above Iron Gate Dam in the Klamath River and upstream habitats (Behnke 1992). Sampling of *O. mykiss* in the mainstem Klamath River (i.e., the Keno Reach) and Upper Klamath Lake revealed body lengths (mean ± SD) of 355 ± 48 mm (Hahlbeck et al. 2023) and 549 ± 47 mm (Hahlbeck et al. 2021), respectively. These body sizes are much larger than those of resident *O. mykiss* rearing in tributary streams (Al Chokhachy et al. 2022; Benjamin et al. 2023). Thus, tributary streams that harbor spawning runs of larger‐bodied fish were designated as fluvial life histories if they drained into Upper Klamath River, and adfluvial life histories if they drained into Upper Klamath Lake. We designated collections as being dominated by resident life histories if large‐bodied *O. mykiss* were not observed there. Prior radio telemetry work corroborated these designated life histories (Hahlbeck et al. 2021). While designated life history types likely represent most samples within each collection, we acknowledge that there may be variation in life history type within collections (alpha diversity) as well as between collections (beta diversity) (Shida and Sato 2025). For anadromous, fluvial, and resident life history types, we classified fish < 70 mm as juveniles and > 200 mm as adults. Fish with FL measurements between 70 and 200 mm were classified as unknown. For the adfluvial life history type, we classified fish < 200 mm as juveniles and > 200 mm as adults.

### 2.2 Genotyping, Quality Filtering, and Sampling Collections

Genomic DNA was extracted from fin tissue following the methods of Ivanova et al. (2006). Samples were genotyped at a panel of previously described SNPs using the Genotyping‐in‐Thousands by sequencing method (GT‐seq) (Campbell et al. 2015). The GT‐seq SNP panel used in this study was developed by the Columbia River Inter‐Tribal Fish Commission and consists of 391 markers, including 251 presumably neutral markers, 135 putatively adaptive markers, four species‐diagnostic markers and one sex identification marker (Collins et al. 2020). Most markers were developed for genetic stock identification, phylogeographic studies, and pedigree reconstruction and were identified in numerous studies (Sprowles et al. 2006; Aguilar and Garza 2008; Sánchez et al. 2009; Abadía‐Cardoso et al. 2011; Campbell et al. 2012; Limborg et al. 2012). The presumably neutral markers exhibit high diversity within or differentiation among broad geographic areas while the putatively adaptive markers were identified from environmental association analyses, or demonstrated strong associations with ecologically relevant phenotypic variation (Pearse et al. 2014; Hess et al. 2016; Micheletti, Hess, et al. 2018a; Micheletti, Matala, et al. 2018b).

We filtered individuals and markers iteratively to remove those with poor quality based on genotyping call rates and a measure of cross contamination (i.e., the “individual fuzziness index,” IFI; Campbell 2017). Quality filtering followed the stepwise scheme detailed in Dayan et al. (2023), and after the final iteration all individuals had an IFI score ≤ 2.5, were genotyped at ≥ 90% of markers, and all markers were genotyped in ≥ 80% of individuals. We removed putative paralogous sequence variants and markers that were monomorphic across all samples. We then identified and removed duplicate tissue samples which can arise during batch sampling with Prevosti's absolute genetic distance in the R4.3.1 package POPPR (Kamvar et al. 2014; R Core Team 2023). Finally, we removed a single potential hybrid that was heterozygous at one species‐specific identification marker (Ocl_gshpx‐357).

Following quality filtering, locations containing fewer than ten individuals were documented. To retain samples in these instances, we pooled individuals into a single collection when they were collected less than 8 km apart in tributaries of the same watershed and were the same life history type. Typically, individuals sampled from the same location across multiple years in contemporary collections (i.e., 2015–2019) were also pooled. However, given that there are two distinct spawning peaks (January and May; ODFW 2016) of adfluvial *O. mykiss* in Spring Creek (a tributary of the Williamson River), juvenile samples from the two collections were not pooled and we tested for genetic differences within a single year. Details on sampling dates and locations are available in the supplement (Table S1).

### 2.3 Presumably Neutral and Putatively Adaptive Marker Datasets

Our analyses focused on two datasets: (1) presumably neutral markers and (2) putatively adaptive markers, including those that map to the chromosomal inversion (Omy5) region associated with anadromy/residency (Pearse et al. 2014; Pearse, Barson, et al. 2019a) and to the chromosome 28 (Omy28) *greb1l*—*rock1* region associated with adult migration timing (reviewed by Waples et al. 2022). Details on the genomic position, primer information, type (i.e., presumably neutral or putatively adaptive) and source of the markers are available in the supplement (Table S2).

In the presumably neutral marker dataset, we identified and filtered markers that did not conform to Hardy–Weinberg (HWE) and linkage equilibrium at the watershed level of the sampling hierarchy. This approach was used to assess HWE and linkage disequilibrium (LD) in a filtering scheme intended to balance the drawbacks of filtering at the site or basin levels of the hierarchy (Pearman et al. 2022). Specifically, we identified and removed markers that were out of HWE in six or more watersheds following correction for multiple comparisons with the false discovery rate using the package PEGAS (Paradis 2010). We then identified marker pairs in significant LD in six or more watersheds with the package DARTR (Gruber et al. 2018) and thinned these pairs by removing the marker with the lesser genotyping call rate.

### 2.4 Genetic Diversity and Population Structure

We assessed genetic diversity within all collections sampled from 11 watersheds of the Lower and Upper Klamath River Basin and the Great Basin (Tables 1, 2; Figure S1). Specifically, Nei's expected heterozygosity was quantified using the neutral marker dataset in the package POPPR (Kamvar et al. 2014). We evaluated genetic variation among contemporary collections using Correspondence analysis (CA) in the ADEGENET package (Jombart 2008; Jombart and Ahmed 2011). Population‐level ordinations were conducted using the presumably neutral and putatively adaptive marker datasets as well as the combined dataset.

We used the neutral marker dataset to investigate the most likely number of genetically distinct clusters (*K*) or populations in the contemporary dataset with the Bayesian clustering method implemented in STRUCTURE 2.3.4 (Pritchard et al. 2000). Without making a priori assumptions about population membership, we applied the admixture model that assumes gene flow among populations and allows for correlated allele frequencies across clusters. We performed 10 replicates for each *K* from 1 to 11. All runs had a burn‐in of 10,000 iterations followed by a run length of 20,000 iterations. Results from replicated runs of each *K* were combined using CLUMPAK (Kopelman et al. 2015). We estimated the best supported *K* with *ΔK* and the mean log probability (Figure S2), but given uneven sample sizes and the potential for meaningful low‐level differentiation in our dataset, we report results across two *K* clusters (Cullingham et al. 2020).

We used the neutral marker dataset to estimate genetic differentiation between contemporary collections (Figure S3). Genetic differentiation was quantified using Nei's pairwise *F*_ST_ in the package HIERFSTAT (Goudet et al. 2022).

### 2.5 Genetic Diversity of Adaptive Anadromy/Residency and Adult Migration Timing Markers

We examined differences in genotype frequencies across contemporary collections at six Omy5 markers associated with anadromy/residency phenotypes. To infer phenotypes, we compared genotypes in our dataset to those associated with anadromy/residency across watersheds in Southern Oregon and Northern California (Pearse et al. 2014; Pearse et al. 2019b; Table S3). We characterized genetic variation throughout the Klamath River Basin based on the six Omy5 markers by conducting a population‐level ordination and generating bar plots to summarize genotype frequencies within each collection. Given the strong linkage in the Omy5 region and to simplify the presentation of results, we also generated bar plots based on one representative marker, Omy5‐24,370. We selected this marker because it is in the middle of the linkage block and is highly concordant with the two adjacent markers (OmyR14589 and OmyR33562).

We also examined differences in genotype frequencies across contemporary collections at seven Omy28 markers associated with adult migration timing. To infer phenotypes, we compared genotypes in our dataset to those associated with early‐ and late‐migration in Rogue River, Oregon *O. mykiss* (Dayan et al. 2023). We characterized genetic variation throughout the Klamath River Basin based on the seven Omy28 markers by conducting a population‐level ordination and generating bar plots to summarize genotype frequencies within each collection. To simplify presentation of the results, we also generated bar plots based on one representative marker, OmyRAD15709‐53, which has previously been shown to have a strong association with adult migration timing in *O. mykiss* from the nearby Rogue River, Oregon (Dayan et al. 2023).

### 2.6 Temporal Genetic Differentiation

Genetic differentiation over time was evaluated in 13 locations with corresponding past (i.e., 2000–2006) and contemporary (i.e., 2016–2019) collections (Table 3). Temporal genetic differentiation was quantified separately with both the presumably neutral and putatively adaptive marker sets using Nei's pairwise *F*_ST_ in the R package HIERFSTAT (Goudet et al. 2022). For each pairwise estimate, the 95% confidence interval was estimated by bootstrapping over markers with 1000 resamples. We considered estimates of genetic differentiation significant when their 95% confidence intervals did not overlap with zero. Results for the Omy5‐24370 and OmyRAD15709‐53 markers are also presented separately to specifically summarize temporal changes in the prevalence of genotypes associated with anadromy/residency and adult migration timing, respectively.

## 3 Results

### 3.1 Genotyping

Quality filtering, pooling of collections, and HWE/LD thinning (i.e., of the neutral marker dataset only) resulted in 2466 *O. mykiss* samples from 74 collections genotyped at 193 presumably neutral markers and 105 putatively adaptive markers. Median base pair read depth per individual and marker was 153 with a mean of 298 ± 474 (SD). Contemporary collections (*n* = 61) sampled in the years 2015–2019 included 2081 individuals from 60 locations within 11 watersheds of the Upper and Lower Klamath River Basin and the Great Basin. The four species‐diagnostic markers and one sex marker were not included in the subsequent analyses.

### 3.2 Genetic Diversity Within and Among Contemporary Collections

Across all 61 contemporary collections, expected heterozygosity based on the presumably neutral marker dataset ranged from 0.07 in Foster Creek (FOST) to 0.30 in Eltapom/Hyampom (ELTP) and Whitworth creeks (WHIT) (Tables 1, 2). On average, 47 markers were monomorphic within each contemporary collection. Yet, in Foster Creek, 152 of the 193 markers were monomorphic, further indicating low genetic diversity in this collection. While the sample size for Foster Creek was small (*n* = 13), other collections with the same sample size had far fewer monomorphic markers (e.g., Grouse Creek has *n* = 13 and 30 monomorphic markers). Within each of the 11 watersheds, expected heterozygosity ranged from 0.13 in the Wood River to 0.29 in the Trinity River. Correspondingly, mean expected heterozygosity was higher among collections below Upper Klamath Lake (*H*_E_ = 0.28) compared to above (*H*_E_ = 0.22).

A comparison across the most prevalent life history types within each collection revealed that expected heterozygosity was significantly greater in collections of anadromous *O. mykiss* compared with collections of adfluvial, fluvial, and resident fish occupying upstream localities and was not associated with sample size (ANCOVA on ranks; df = 3; *F* = 25.8; *p* < 0.001; Figure S1). Conversely, expected heterozygosity was significantly lower in collections of adfluvial *O. mykiss* compared with collections of anadromous, fluvial, and resident fish. Lastly, expected heterozygosity was not significantly different when comparing fluvial and resident collections.

### 3.3 Genetic Structure

Ordinations representing genetic variation among contemporary collections based on the presumably neutral, putatively adaptive, and combined marker datasets were concordant and supported a division between coastal and inland *O. mykiss* lineages located primarily downstream and upstream of the outlet of Upper Klamath Lake, respectively (Figure 2, Figure S4a,b). The divide between the two lineages can be envisioned as a vertical line through dimension 1, between −0.25 and 0, with the coastal lineage on the left and the inland lineage on the right. Dimension 1 grouped collections into two main clusters. The first cluster consists of primarily anadromous and resident *O. mykiss* in downstream locations of the Trinity, Salmon, and Klamath rivers but also resident fish from two of the Klamath Lake Cascade Tributaries collections, Denny (DENN) and Cherry (CHER) creeks. The second cluster consists of mainly adfluvial individuals of the Wood and Williamson rivers but also includes adfluvial individuals from Kamkaun Spring (KAMK) and Beatty Gap (BTTY) of the mainstem Sprague River where groundwater springs are located. Most collections of resident and fluvial *O. mykiss* from the Sprague River tributaries as well as the Upper Williamson River (UWIL) and Moss Creek (MOSS) collections fell intermediately between the two primary clusters. There was little genetic difference between summer (SCSU) and fall (SCFA) collections within Spring Creek in the Williamson River based on the neutral or adaptive markers. Of the two Great Basin collections, Chewaucan River (CHEW) was distinct from all other collections along dimensions 1 and 2 while Foster Creek clustered with adfluvial *O. mykiss* . Lastly, fish sampled at the Klamath Hatchery (Oak Springs Stock 53) (KHAT) were distinguishable from all natural collections along dimension 2.

**FIGURE 3:**
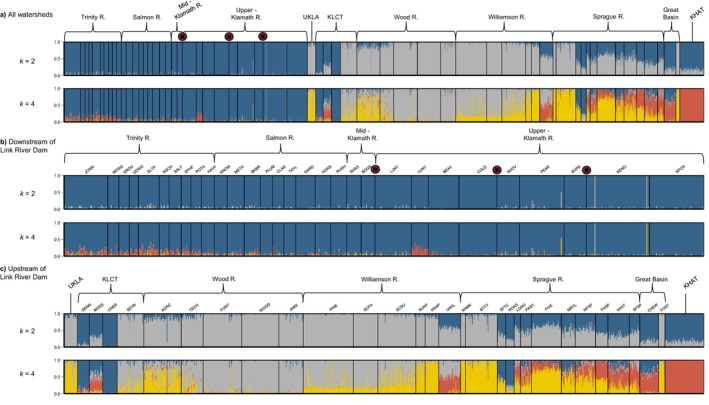
Admixture proportions estimated with the neutral marker dataset and *K* = 2 or *K* = 4 clusters in the program STRUCTURE. Samples include *O. mykiss* from contemporary collections (*n* = 2081 individuals in 61 collections) across (a) all watersheds, (b) watersheds downstream of the outlet of Upper Klamath Lake, and (c) Upper Klamath Lake and upstream watersheds. *K* = 2 was the best supported number of clusters. Dams removed after sample collection are indicated by black squares within red circles, from left to right within (a) and (b): Iron Gate Dam, Copco 1 and 2 Dams, and J.C. Boyle Dam.

Evaluation of STRUCTURE results for the contemporary collections indicated that *K* = 2 was the best supported number of *K* clusters (Figure S2). Results from an unreported analysis that incorporated the eight markers filtered due to Hardy–Weinberg equilibrium also identified two significant *K* clusters and did not differ qualitatively in patterns of admixture. Additionally, results with *K* = 4 clusters were evaluated to identify any low‐level patterns of differentiation. In analyses with both *K* = 2 and *K* = 4 clusters, there was a divide in the genetic composition of *O. mykiss* downstream and upstream of Upper Klamath Lake representing the coastal and inland lineages, respectively (Figure 3a). Downstream of the lake in the Trinity, Salmon, and Klamath rivers, *O. mykiss* were genetically similar (Figure 3b). Moreover, there was little genetic differentiation among sampling locations separated by Iron Gate Dam, Copco Dams 1 and 2, or J.C. Boyle Dam in the Mid‐ and Upper‐Klamath River. Upstream of Upper Klamath Lake, *O. mykiss* collections were genetically diverse. Locations within the Klamath Lake Cascade Tributaries (KLCT) harbored fish with coastal, inland, and admixed genetic signatures despite being in close geographic proximity (Figure 3c). For instance, *O. mykiss* sampled in Denny (DENN) and Cherry (CHER) creeks resembled downstream collections representing the coastal lineage, whereas those sampled in Sevenmile Creek (SEVN) had an inland genetic signature, and individuals from Moss Creek (MOSS) were admixed. With some exceptions, individuals sampled in the Wood and Williamson rivers exhibited inland genetic compositions. However, despite being located upstream of Williamson River Falls and Klamath Marsh, fish in the Upper Williamson River (UWIL) displayed admixed proportions of both coastal and inland signatures. Collections from the Sprague River exhibited either admixed (SFTC, ROKS, LONG, PASY, FIVE, MRYL, NFSP, PASP, WHIT, SFSP) or predominantly inland genetic signatures (KAMK, BTTY). Of the two Great Basin collections, the Chewaucan River (CHEW) exhibited a primarily coastal genetic signature while Foster Creek (FOST) displayed an inland signature. Similar to the Chewaucan River (CHEW), the Klamath Hatchery (KHAT) exhibited a predominantly coastal lineage genetic signature.

Pairwise estimates of genetic differentiation based on the neutral marker dataset were consistent with the ordination and Bayesian clustering analysis results (Figure S3). For instance, collections below and above the outlet of Upper Klamath Lake were genetically differentiated with the few exceptions as noted above. Below the lake, collections were genetically similar while the heatmap captured the mosaic pattern of genetic differentiation for collections above the outlet of Upper Klamath Lake.

### 3.4 Genetic Diversity at the Adaptive Anadromy/Residency Markers

**FIGURE 4:**
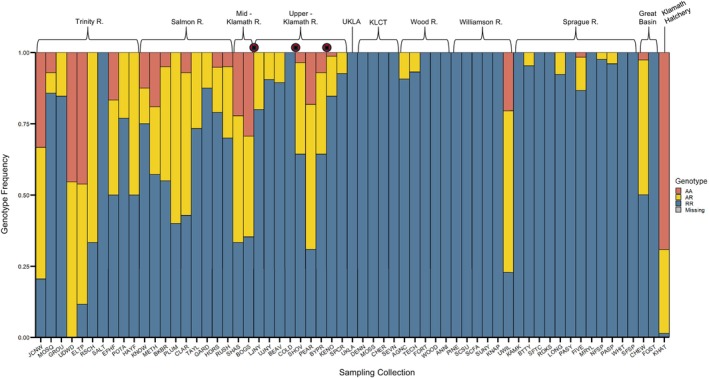
*O. mykiss* genotype frequencies across contemporary collections (*n* = 2081 individuals from 61 collections within 11 watersheds) at a representative Omy5 marker associated with anadromy/residency phenotypes (Omy5‐24370). Colors indicate genotypes homozygous for alleles associated with anadromy (AA), heterozygous genotypes (AR), genotypes homozygous for alleles associated with residency (RR), and missing genotypes. Dams removed after sample collection are indicated by black squares within red circles, from left to right: Iron Gate Dam, Copco 1 and 2 Dams, and J.C. Boyle Dam.

**FIGURE 5:**
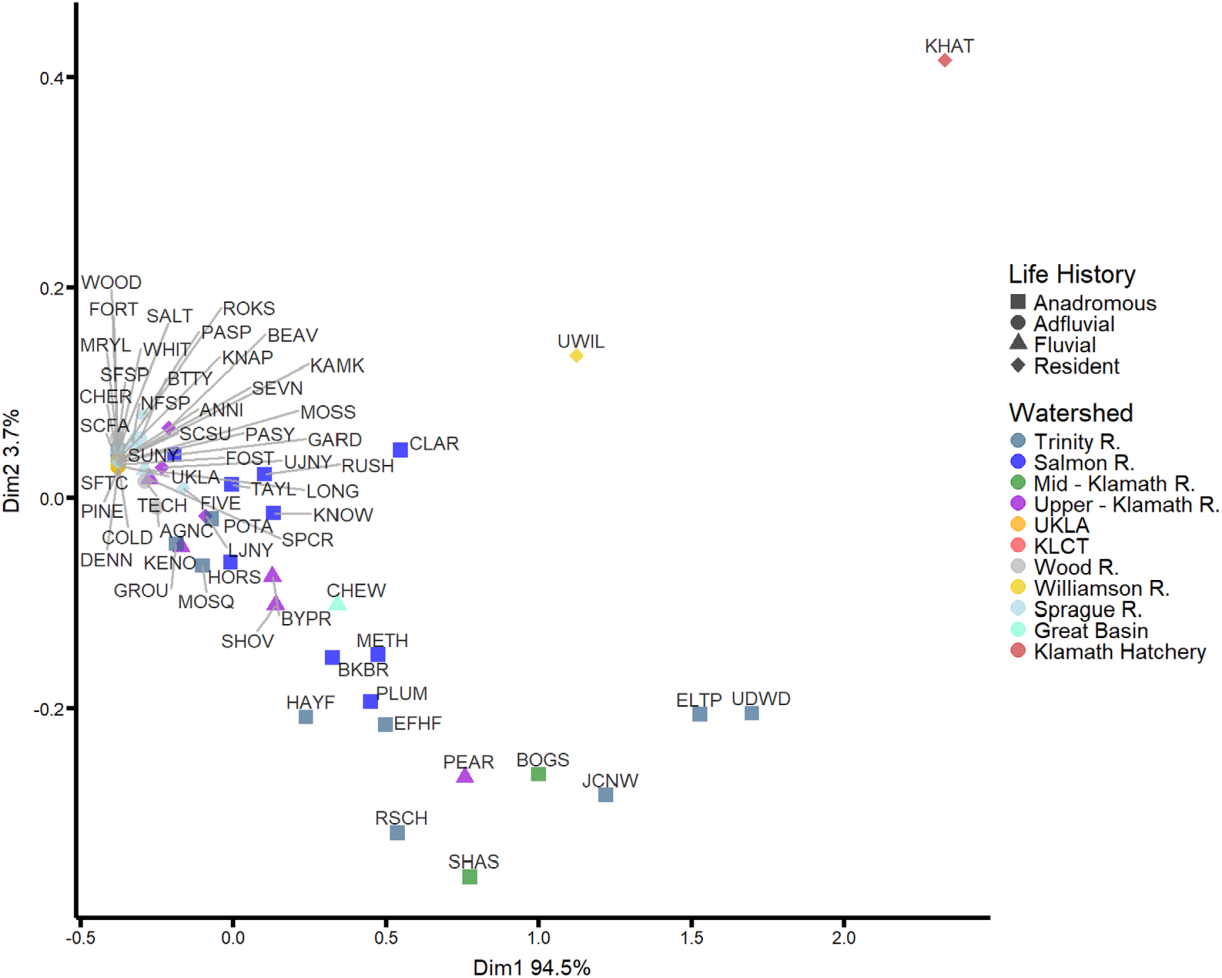
Population‐level ordination of *O. mykiss* contemporary collections (*n* = 2081 individuals from 61 collections within 11 watersheds) based on the six Omy5 markers associated with anadromy/residency phenotypes. Shape and color of data points indicate the most prevalent life history type and watershed for each collection, respectively.

Genotype frequencies at the Omy5‐24370 marker in the Omy5 region associated with anadromous/resident phenotypes in *O. mykiss* further emphasized the distinction between contemporary collections upstream and downstream of Upper Klamath Lake (Figure 4). Downstream of the lake, 11 of the 30 collections contained 50% or greater anadromous/heterozygous genotypes. Two collections (SALT and COLD) were fixed for the resident genotype. In contrast, Upper Klamath Lake (UKLA) and most of the upstream collections were entirely fixed for resident genotypes while the Upper Williamson (UWIL), Chewaucan (CHEW), and the Klamath Hatchery (KHAT) harbored a mixed assemblage of anadromous, resident, and heterozygous genotypes. Seven other collections exhibited a low frequency of anadromous/heterozygous genotypes (AGNC, TECH, BTTY, LONG, FIVE, NFSP, PASP). Variation in genotype frequency at Omy5‐24370 was supported by four other Omy5 markers, with only one, OmyR40319, at times discordant from the others, particularly in collections downstream of Upper Klamath Lake (Figure S5). The ordination based on all six Omy5 markers also highlights the variation in genotypic frequency among collections while designating life history type (Figure 5).

### 3.5 Genetic Diversity at the Adaptive Adult Migration Timing Markers

**FIGURE 6:**
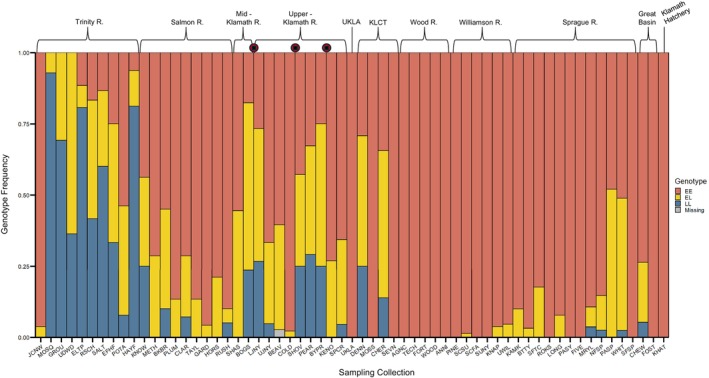
*O. mykiss* genotype frequencies across contemporary collections (*n* = 2081 individuals from 61 collections within 11 watersheds) at a representative Omy28 marker associated with adult migration timing (OmyRAD15709‐53). Colors indicate early‐migration timing associated homozygous genotype (EE), heterozygous genotype (EL), late‐migration timing associated homozygous genotype (LL), and missing genotypes. Dams removed after sample collection are indicated by black squares within red circles, from left to right: Iron Gate Dam, Copco 1 and 2 Dams, and J.C. Boyle Dam.

**FIGURE 7:**
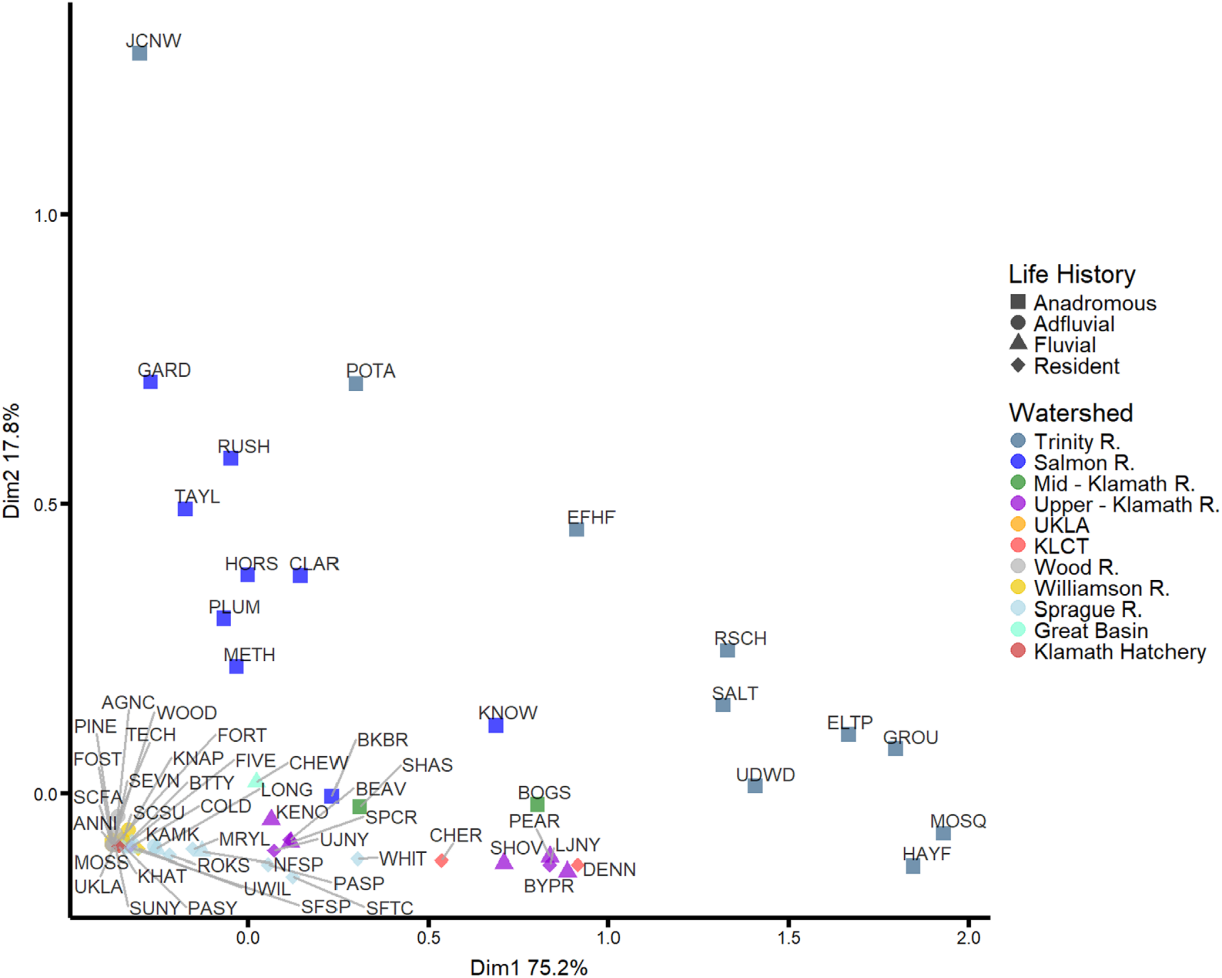
Population‐level ordination of *O. mykiss* contemporary collections (*n* = 2081 individuals from 61 collections within 11 watersheds) based on the seven Omy28 markers associated with adult migration timing. Shape and color of data points indicate the most prevalent life history type and watershed for each collection, respectively.

Similarly, genotype frequencies at the *greb1l–rock1* region marker OmyRAD15709‐53 on chromosome 28 associated with adult migration timing highlighted the difference between contemporary collections upstream and downstream of Upper Klamath Lake (Figure 6). Downstream of the lake, 14 of the 30 collections contained 50% or greater late‐migration timing/heterozygous genotypes. Three collections were nearly fixed for the early‐migration genotype (JCNW, GARD, COLD). In contrast, Upper Klamath Lake (UKLA) and the majority of the upstream collections were entirely fixed for the early‐migration genotype. Denny (DENN), Cherry (CHER), Paradise (PASP) creeks contained 50% or greater late‐migration timing/heterozygous genotypes. Twelve other collections exhibited a low frequency of late‐migration timing/heterozygous genotypes (SCSU, KNAP, UWIL, KMK, BTTY, SFTC, LONG, MRYL, NFSP, PASP WHIT, CHEW). Patterns in genotype frequency at OmyRAD15709‐53 were supported by five other markers in the *greb1l–rock1* region (Figure S6). However, Chr28_11658853 was often discordant with the others, especially in collections upstream of Upper Klamath Lake. The ordination based on all seven Omy28 markers also highlights the variation in genotypic frequency among collections while designating life history type (Figure 7).

### 3.6 Temporal Genetic Differentiation

Of the 13 locations with corresponding past and contemporary collections, nine exhibited significant temporal genetic differentiation based on the neutral or adaptive marker datasets over the approximate 15‐year period (Table 3). Three of these comparisons were located below the outlet of Upper Klamath Lake while the other six were located above the outlet. Estimates of genetic differentiation in these collections had a maximum *F*_ST_ of 0.104 ± 0.060 in Rock Creek (ROKS) of the Sprague River based on the adaptive marker dataset. Correspondingly, this collection also had the maximum estimated genetic differentiation based on the neutral marker dataset (0.084 ± 0.034), indicating moderate temporal shifts in allele frequency over the timeframe evaluated. When comparing the neutral and adaptive marker datasets, we did not detect a consistent difference in the magnitude patterns of genetic differentiation. Nonetheless, *F*_ST_ in the adaptive marker dataset was six, three, and two times that of the neutral dataset for Long (LONG), Methodist (METH), and Cherry (CHER) creeks, respectively.

Lastly, we detected little or no temporal variation in genotype frequencies at the Omy5 marker and *greb1l–rock1* region marker OmyRAD15709‐53 in 12 of the 13 locations with corresponding past and contemporary collections (Figure S7). Notable shifts for both adaptive regions were detected only in Methodist Creek (METH) of the Salmon River. In the past collection, genotype frequencies at Omy5‐24370 were approximately half homozygous resident and half heterozygous. In the contemporary collection, half remained homozygous resident while approximately a quarter remained heterozygous and the remaining quarter were homozygous anadromous. Similarly, there was a shift in the Methodist Creek (METH) collection at OmyRAD15709‐53. In the past collection, half were late‐migration genotypes, roughly a quarter heterozygous and a quarter early‐migration genotype. In the contemporary collection, no late‐migration genotypes were detected. Instead, the majority were early‐migration genotypes and approximately a quarter were heterozygous.

## 4 Discussion

We present a comprehensive genome‐wide evaluation of genetic diversity and structure of coastal and inland *O. mykiss* in the Klamath River Basin prior to dam removal, as well as in the neighboring Great Basin using both presumably neutral and putatively adaptive genetic markers. Given that access to the upper Klamath watershed has been restored, this study will serve as a critical baseline for future genetic monitoring efforts focusing on changes in *O. mykiss* population structure as well as shifts in genotypic frequency at markers associated with anadromy/residency (*Omy5*) and adult return timing (*Omy28*).

### 4.1 Genetic Structure Within and Among Contemporary Collections

We found a clear division between coastal and inland *O. mykiss* lineages located primarily downstream and upstream of the outlet of Upper Klamath Lake, respectively, using complementary population genetic analyses. First, ordinations based on the presumably neutral, putatively adaptive, and combined marker datasets separated the anadromous and resident *O. mykiss* downstream of the Upper Klamath Lake from primarily adfluvial fish sampled in the Wood, Williamson, Sprague, and KLCT watersheds. Notably, two collections of resident fish from the KLCT watershed (DENN and CHER creeks) clustered with the coastal lineage downstream of the lake. This finding could be attributed to three different mechanisms: (1) gene flow from upper Klamath River *O. mykiss* via the Link River Dam fish ladder, (2) remanent steelhead, or the most likely (3) historical fish stocking when Spencer Creek Hatchery was operational. Second, the Bayesian clustering analysis implemented in STRUCTURE also showed a clear division between the coastal *O. mykiss* lineage downstream of the outlet of Upper Klamath Lake and the inland lineage located upstream, except for the two KLCT collections. While we detected two significant clusters, we caution that interpretation of the optimal *K* in our study may be complicated by life history differences, variation in admixture due to historical stocking, as well as unequal sample sizes across collections (Janes et al. 2017). Nonetheless, the primary division between inland and coastal lineages has been documented in previous studies using allozyme (Buchanan et al. 1994; Currens et al. 2009) and microsatellite (Pearse et al. 2011) data and demonstrates that despite the constraints imposed on fish migrations by the construction of four dams, there was no significant differentiation among collections in the Upper Klamath River watershed.

Both the ordination and Bayesian clustering analyses used here also captured the mosaic genetic patterns that exist upstream of the outlet of Upper Klamath Lake. For instance, resident and fluvial *O. mykiss* located in Sprague River tributaries as well as the Upper Williamson River (UWIL), Moss Creek (MOSS), and Klamath Hatchery (KHAT) collections show an association with the Klamath River coastal genetic lineage. Of the two Great Basin collections, resident *O. mykiss* from Foster Creek (FOST) grouped with the adfluvial fish and were almost exclusively inland lineage whereas fluvial *O. mykiss* from the Chewaucan River (CHEW) were distinct from all collections based on the ordination and are predominantly coastal lineage. Historically, the Chewaucan River was stocked with hatchery rainbow trout with origins likely from the Sacramento River, which is also where the Klamath Hatchery stock used in this analysis originated. The presence of inland, coastal, and admixed genetic lineages upstream of the Upper Klamath Lake outlet supports the hypothesis that an ancestral lineage of *O. mykiss* which was isolated in the upper basin was secondarily invaded by the *O. mykiss* coastal lineage when the Klamath River connected with Upper Klamath Lake and provided an outlet to the Pacific Ocean (Currens et al. 2009; Pearse et al. 2011).

### 4.2 Genetic Diversity Within and Among Contemporary Collections

We found that genetic diversity, as measured by expected heterozygosity, was lowest in the Great Basin (FOST) and highest in collections both below (ELTP) and above (WHIT) Upper Klamath Lake. Overall, genetic diversity was slightly higher in collections below compared to collections upstream of the Upper Klamath Lake outlet. Using the life history type designation, which again represents most samples within each collection, we found that genetic diversity was significantly greater in anadromous *O. mykiss* collections compared with adfluvial, fluvial, and resident collections while genetic diversity was significantly lower in collections of adfluvial *O. mykiss* compared with anadromous, fluvial, and resident collections. While the finding for adfluvial collections could potentially be attributed to sampling young of the year with high relatedness, it is more likely attributed to the limited number of effective adfluvial spawners within each collection given that the genetic diversity of adfluvial adults sampled within Upper Klamath Lake (UKLA) was also low. Furthermore, a similar finding has been reported for adfluvial fish in a smaller survey (DeHann and Von Bargen 2015).

### 4.3 Genetic Diversity at the Adaptive Anadromy/Residency Markers

Consistent with our results based on the neutral marker dataset, *O. mykiss* collections located downstream and upstream of the outlet of Upper Klamath Lake were differentiated based on variation at the anadromy/residency and adult migration timing markers. Focusing first on the chromosome Omy5 markers associated with anadromous/resident phenotypes, we found that anadromous and heterozygous genotypes were more prevalent downstream of the outlet and this pattern was largely consistent across five of the six markers. OmyR40319 is located at the end of the linkage block which likely explains the high discordance as well as the higher rate of missing data. Upstream of the outlet of Upper Klamath Lake, resident Omy5 genotypes were dominant except in three collections (UWIL, CHEW, KHAT). Interestingly, resident *O. mykiss* in the Upper Williamson River were primarily anadromous or heterozygous across all six Omy5 markers; a result consistent with Pearse et al. (2019b). One possible explanation is that coastal steelhead historically ascended Williamson River falls during favorable flows, and that this population has retained the standing variation at Omy5 associated with anadromy. Alternatively, the results could be influenced by historical hatchery stocking of *O. mykiss* that originate from a coastal lineage such as the Sacramento River. Nonetheless, our population genetic analyses provide support for coastal origin of *O. mykiss* in the Upper Williamson River. Similarly, *O. mykiss* from the Chewaucan River (CHEW) and Klamath Hatchery (KHAT) are of coastal lineage and have primarily anadromous/heterozygous genotypes, which may result in part from the stocking of Sacramento River origin fish. Despite extensive migrations made by adfluvial fish in the Upper Klamath River Basin, only two of the 14 adfluvial collections had heterozygous genotypes, Agency (AGNC) and Tecumseh (TECH) creeks, while the remaining were all homozygous resident. These two collections are located downstream of the Klamath Hatchery and may reflect escapees from the hatchery. A common theme in other studies that have identified anadromous‐associated alleles in adfluvial *O. mykiss* is that these populations are generally derived from coastal origin (Leitwein et al. 2017; Arostegui et al. 2019). Our genetic data highlight the strong divergence between coastal *O. mykiss* genetic lineage located downstream of Upper Klamath Lake and the inland genetic lineage located upstream of the outlet of Upper Klamath Lake. This finding supports previous hypotheses proposed by Behnke (2002), Currens et al. (2009), and Pearse et al. (2011) that adfluvial, fluvial, and resident *O. mykiss* in the Upper Klamath River Basin originated from ancient interior connections and had little genetic connectivity with coastal *O. mykiss* after the formation of the Klamath River.

### 4.4 Genetic Diversity at the Adaptive Adult Migration Timing Markers

The results of our geographic survey of variation associated with adult migration timing on the chromosome 28 (Omy28) *greb1l*—*rock1* region were largely concordant with previous studies (Pearse et al. 2019b; Fraik et al. 2021), with strong associations observed between genotype and known population phenotypes. The seven Omy28 markers were mostly concordant except for OmyChr28_11658853, which is located near the end of the linkage block. Discordance at OmyChr28_11658853 was observed in collections upstream of the Trinity River. Downstream of Upper Klamath Lake, late‐migration timing and heterozygous genotypes were numerous while early‐migration timing genotypes were prevalent upstream of the lake outlet. In addition, we found that *O. mykiss* inhabiting tributaries of the Trinity River below Iron Gate Dam had the highest proportions of late‐migration timing genotypes whereas fish inhabiting tributaries of the Salmon River were primarily early‐migration timing with a small proportion of heterozygotes. Consistent with results based on the neutral marker dataset, Cherry (CHER) and Denny (DENN) creeks had proportions of Omy28 genotypes similar to collections below the lake. It is important to note that these results could be influenced by the timing of sample collection, especially for tissue samples collected from adults (Table S1). For example, samples collected from Junction City Weir on the Trinity River were collected from adults in the summer and early fall, missing any late‐migrating adults at this site. Overall, our findings for *O. mykiss* sampled above the Upper Klamath Lake outlet address one of the uncertainties underscored in Narum et al. (2023): *Do resident populations harbor genetic variation for migration timing?* We detected early‐migration timing genotypes in every upstream collection and in many cases, occurring at 100% frequency.

### 4.5 Temporal Genetic Differentiation

Of the thirteen locations examined, we found evidence for significant genetic differentiation between past and contemporary collections in five based on both the neutral and adaptive marker datasets. Three of these were in Sprague River tributaries (SFTC, ROKS, PASY) and the magnitude of differentiation, as estimated by *F*_ST_, was similar for each marker set which could be indicative of genetic drift and/or successful immigration and gene flow into these populations. The remaining two locations in the KLCT (CHER) and the Salmon River (METH) were more differentiated based on the adaptive marker datasets and thus might be driven by selection acting on these populations.

Notable shifts in genotype frequencies at the Omy5 marker and *greb1l*—*rock1* region marker OmyRAD15709‐53 were only detected in Methodist Creek (METH), a tributary of the Salmon River. The location shifted from half resident and half heterozygotes with primarily late‐migration timing genotypes to a quarter anadromous with primarily early‐migration timing genotypes indicating a notable shift in life history diversity. It will be interesting to determine if this trend continues given that the dams have been removed. Genotype frequencies at Omy5‐24370 and OmyRAD15709‐53 in the remaining 12 locations were markedly stable over the ~15‐year timeframe.

### 4.6 Management Approach

Given the complexity and uncertainty surrounding the relationship between resident and anadromous forms of *O. mykiss* in the Upper Klamath River Basin, the Oregon Department of Fish and Wildlife and The Klamath Tribes have adopted a conservative approach to reestablishing coastal steelhead in the upper basin. The prevailing hypothesis is that steelhead in the Klamath River will repopulate habitat upstream of Upper Klamath Lake while resident *O. mykiss* may produce smolts that contribute to the expression of the anadromous life history. As detailed in the reintroduction implementation plan, the adaptive strategy focuses first on allowing natural repopulation via volitional migration of adults. Volitional repopulation will be monitored and evaluated for 15 years (i.e., three generations). Monitoring activities will include snorkel/electrofishing surveys, spawner/carcass surveys, downstream/upstream trapping, mark‐recapture studies, scale analysis, and environmental DNA sampling. A nonlethal fin clip will be collected from every individual for genetic analyses. After this period, an assessment will be conducted to determine if, where, and when active reintroduction is needed to help establish and support the diverse populations of this species in the Klamath watershed (Oregon Department of Fish and Wildlife and the Klamath Tribes 2021).

## Funding

Funding for this project was supported by a grant through the Oregon Department of Fish and Wildlife's Restoration and Enhancement Program. This work was also supported, in part, by the Oregon Agricultural Experiment Station with funding from the Hatch Act capacity funding program, award number NI25HFPXXXXXG022, from the USDA National Institute of Food and Agriculture.

## Conflicts of Interest

The authors declare no conflicts of interest.

## Supporting information


**Figure S1:** Expected heterozygosity across life history types of *O. mykiss* estimated with the neutral marker dataset within contemporary collections (*n* = 2081 individuals in 61 collections). Boxes represent interquartile ranges, horizontal lines are medians, squares are means, and error bars are standard errors. Letters distinguish significant differences among life history types estimated with post hoc multiple comparisons and Tukey's correction.

**Figure S2:** Plots for detecting the best supported number of *K* clusters generated in the program CLUMPAK. In (a) Δ*K*, (b) L'(*K*), and (c) L(*K*) all of which suggest *K* = 2 was the best supported number of clusters.

**Figure S3:** Heatmap of pairwise genetic differentiation (*F*_ST_) among *O. mykiss* contemporary collections (*n* = 2081 individuals from 61 collections). Differentiation was quantified with the neutral marker dataset.

**Figure S4:** Population‐level ordinations of *O. mykiss* contemporary collections (*n* = 2081 individuals from 61 collections within 11 watersheds) based on: (a) the adaptive marker dataset, and (b) the combined marker dataset.

**Figure S5:** eva70297‐sup‐0005‐FigureS5.pptx. *O. mykiss* genotype frequencies across contemporary collections (*n* = 2081 individuals from 61 collections) at six representative Omy5 markers associated with anadromy/residency phenotypes. Colors indicate genotypes homozygous for alleles associated with anadromy (AA), heterozygous genotypes (AR), genotypes homozygous for alleles associated with residency (RR), and uncalled genotypes (Missing).

**Figure S6:** eva70297‐sup‐0006‐FigureS6.pptx. *O. mykiss* genotype frequencies across contemporary collections (*n* = 2081 individuals from 61 collections) at seven representative Omy28 markers associated with adult migration timing. Colors indicate early‐migration timing associated homozygous genotype (EE), heterozygous genotype (EL), late‐migration timing associated homozygous genotype (LL), and uncalled genotypes (Missing).

**Figure S7:** eva70297‐sup‐0007‐FigureS7.pptx. *O. mykiss* genotype frequencies across 13 locations with corresponding past and contemporary collections at representative Omy5 and Omy28 markers associated with anadromy/residency phenotypes and adult migration timing, respectively. In (a) past and (b) contemporary collections colors indicate anadromous associated homozygous genotype (AA), heterozygous genotype (AR), resident associated homozygous genotype (RR), and uncalled genotypes (Missing) at Omy5‐24370. In (c) past and (d) contemporary collections colors indicate early‐migration timing associated homozygous genotype (EE), heterozygous genotype (EL), late‐migration timing associated homozygous genotype (LL), and uncalled genotypes (Missing) at OmyRAD15709‐53.

**Table S1:** Sample collection information. Sample identification, watershed, location, collection date, GPS coordinates, collector, developmental stage, and life history type of collected tissue samples.

**Table S2:** Marker information. Marker identification, genomic position, primer information, presumed type (i.e., neutral or adaptive), and source.

**Table S3:** Genotype to anadromy/residency phenotype associations of Omy5 markers characterized by Pearse et al. (2014).

## Data Availability

The data underlying the main results of this study will be archived in the Dryad Digital Repository following acceptance for publication.
