# Supplementary figures and images for: Characterizing the Distribution of Oncorhynchus mykiss Genetic Diversity in the Klamath River Basin Before Dam Removal
Source: Evol Appl. 2026 Jul 12;19(7):e70297. doi: 10.1111/eva.70297 (PMC13358376; doi:10.1111/eva.70297)

## Slide 1
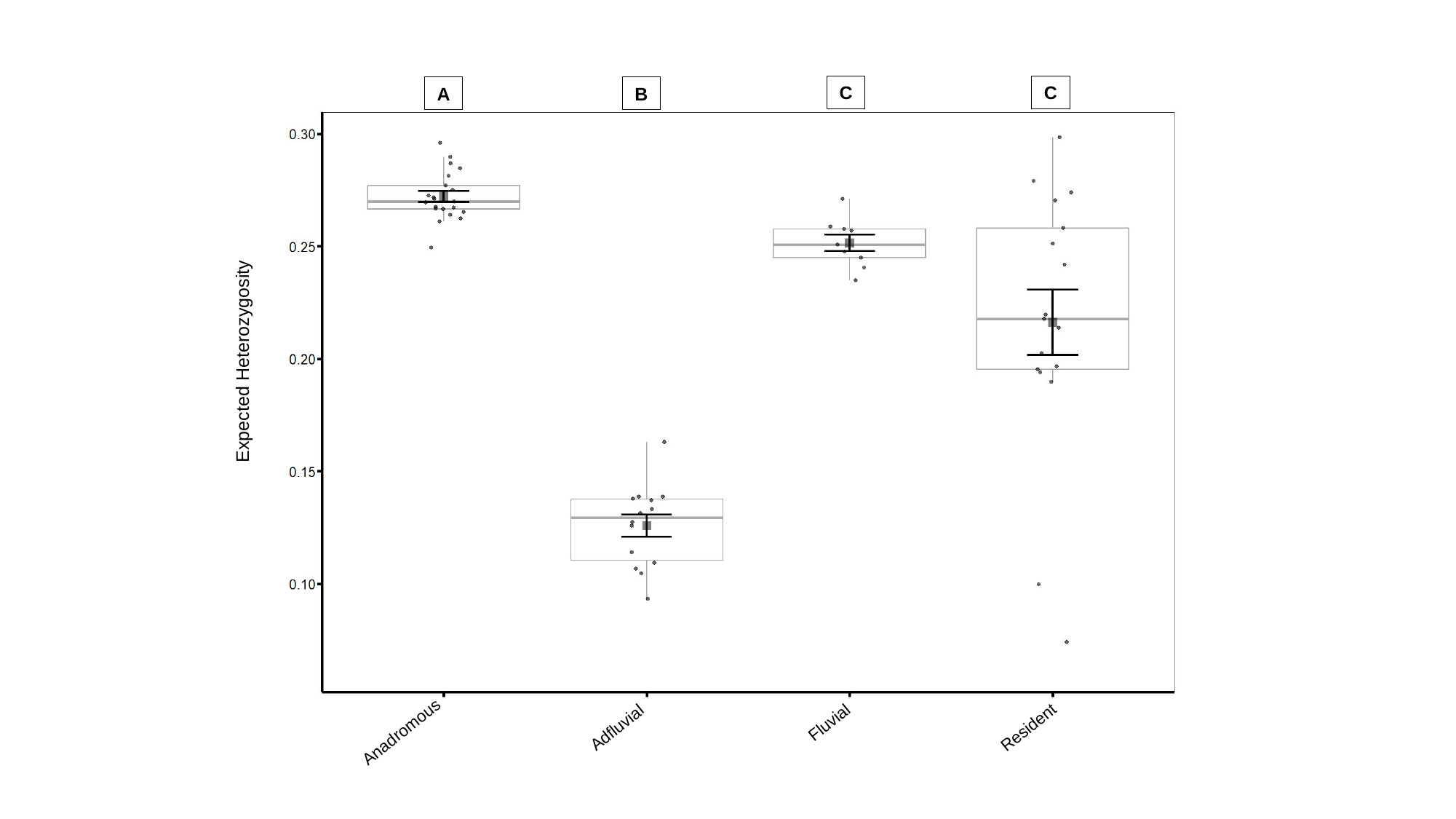

C
C
A
B
 Expected Heterozygosity
Fluvial
Resident
Adfluvial
Anadromous

Supplement: Supplementary file 1 — Figure S1: Expected heterozygosity across life history types of O. mykiss estimated with the neutral marker dataset within contemporary collections (n = 2081 individuals in 61 collections). Boxes represent interquartile ranges, horizontal lines are medians, squares are means, and error bars are standard errors. Letters distinguish significant differences among life history types estimated with post hoc multiple comparisons and Tukey's correction. [file EVA-19-e70297-s008.pptx]

## Slide 1
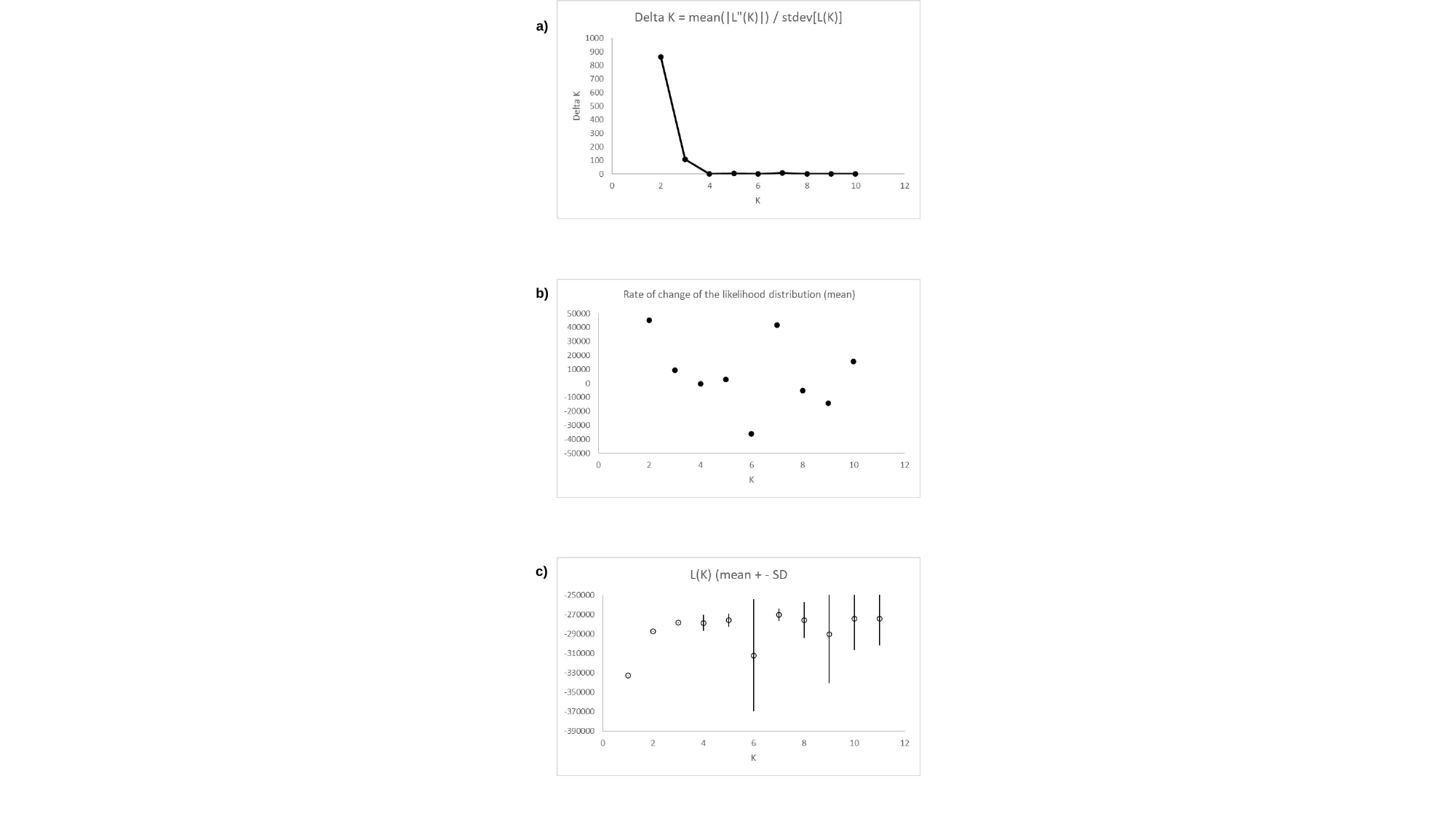

a)
b)
c)

Supplement: Supplementary file 2 — Figure S2: Plots for detecting the best supported number of K clusters generated in the program CLUMPAK. In (a) ΔK, (b) L'(K), and (c) L(K) all of which suggest K = 2 was the best supported number of clusters. [file EVA-19-e70297-s004.pptx]

## Slide 1
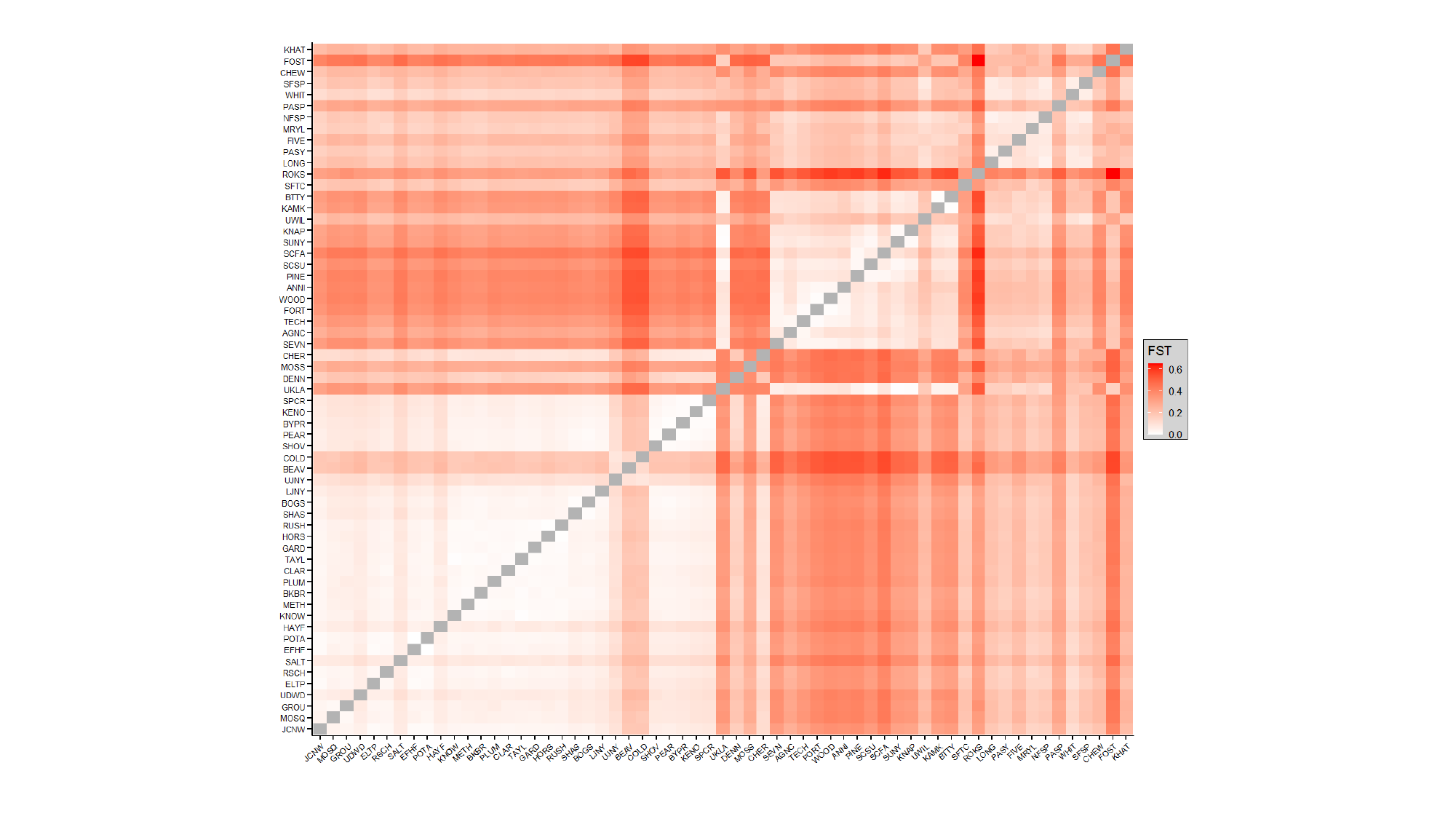

Supplement: Supplementary file 3 — Figure S3: Heatmap of pairwise genetic differentiation (F ST) among O. mykiss contemporary collections (n = 2081 individuals from 61 collections). Differentiation was quantified with the neutral marker dataset. [file EVA-19-e70297-s005.pptx]

## Slide 1
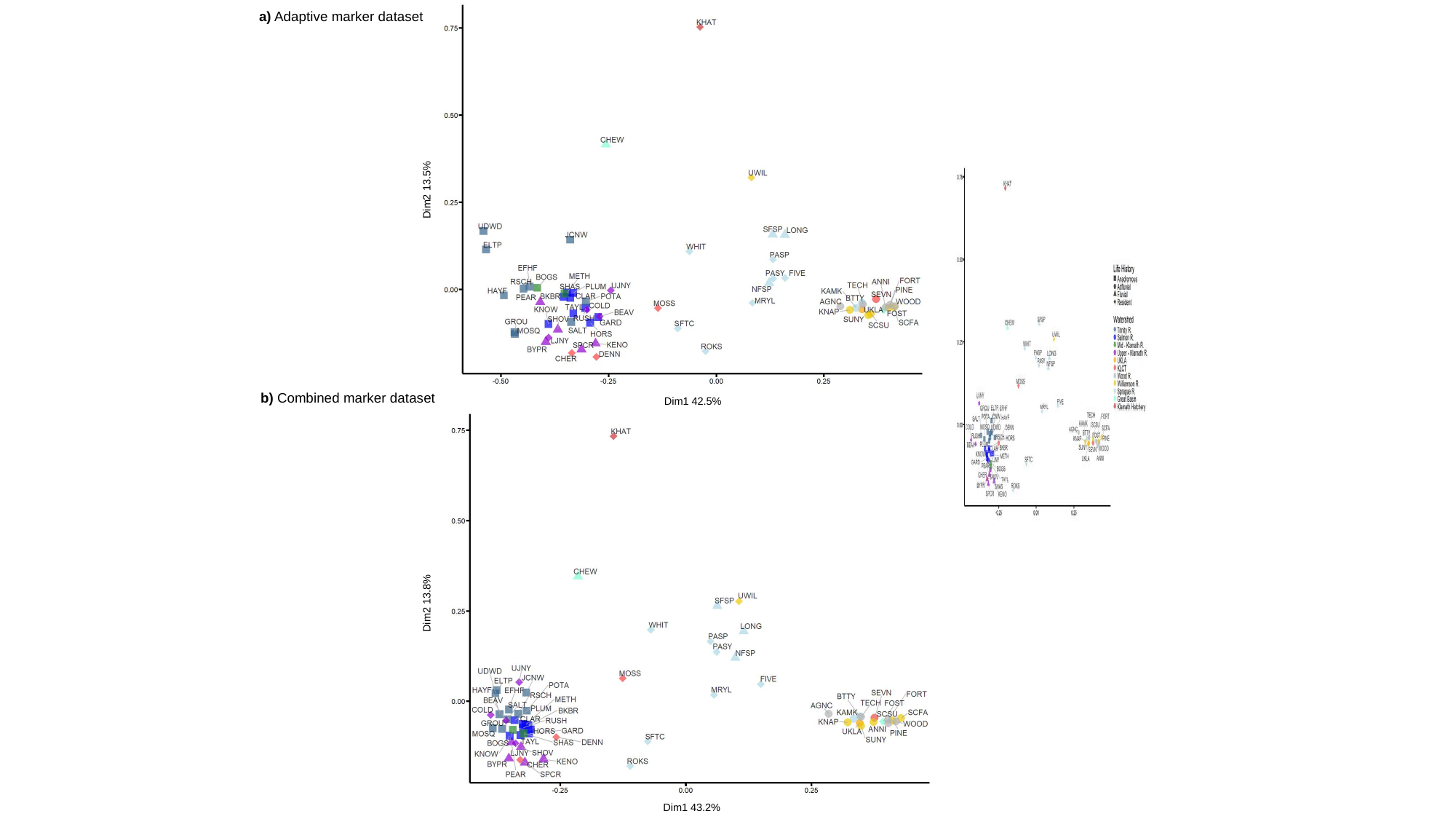

a) Adaptive marker dataset
Dim2 13.5%
b) Combined marker dataset
Dim1 42.5%
Dim2 13.8%
Dim1 43.2%

Supplement: Supplementary file 4 — Figure S4: Population‐level ordinations of O. mykiss contemporary collections (n = 2081 individuals from 61 collections within 11 watersheds) based on: (a) the adaptive marker dataset, and (b) the combined marker dataset. [file EVA-19-e70297-s007.pptx]

## Slide 1
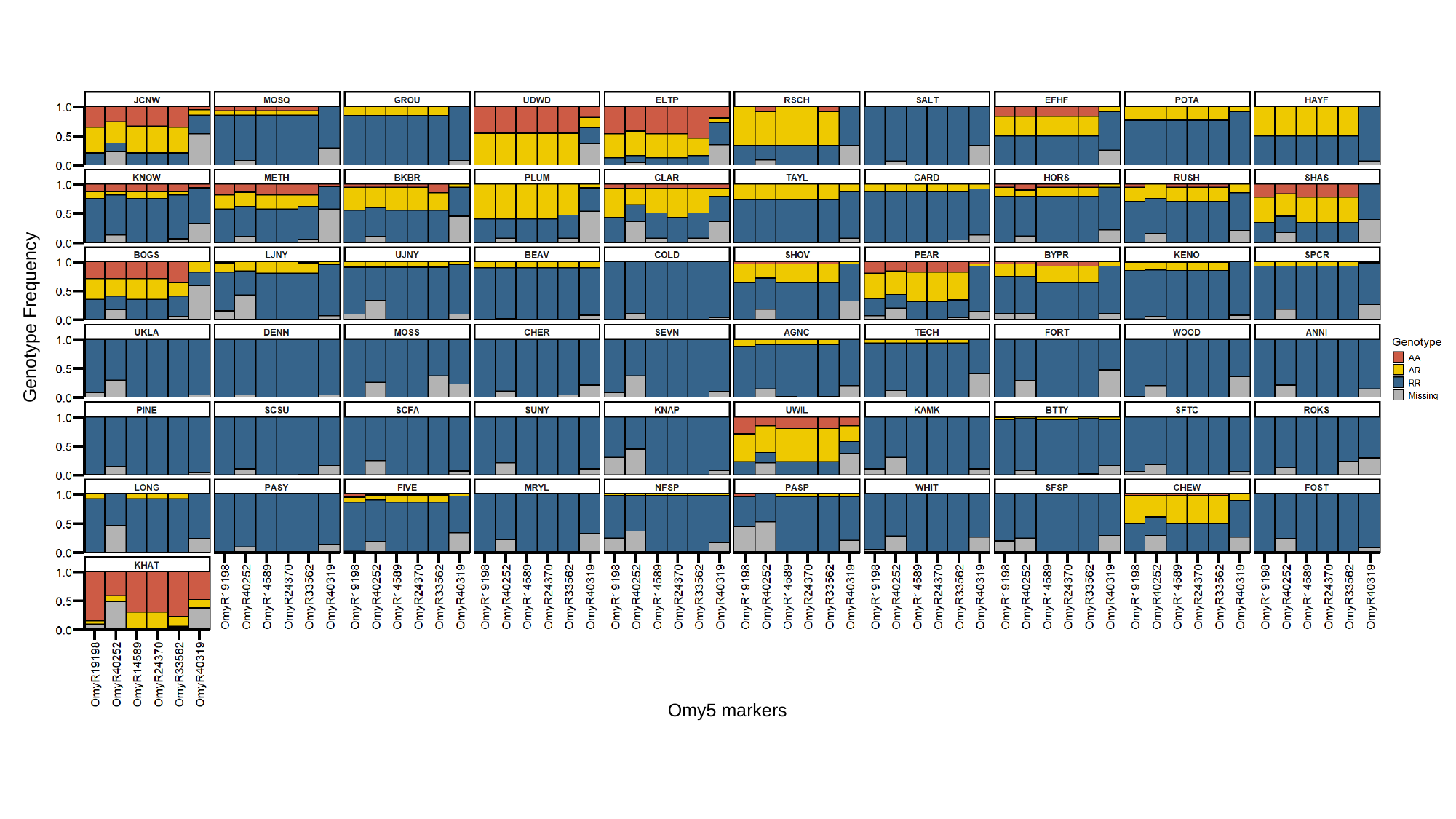

Genotype Frequency
Omy5 markers

Supplement: Supplementary file 5 — Figure S5: eva70297‐sup‐0005‐FigureS5.pptx. O. mykiss genotype frequencies across contemporary collections (n = 2081 individuals from 61 collections) at six representative Omy5 markers associated with anadromy/residency phenotypes. Colors indicate genotypes homozygous for alleles associated with anadromy (AA), heterozygous genotypes (AR), genotypes homozygous for alleles associated with residency (RR), and uncalled genotypes (Missing). [file EVA-19-e70297-s009.pptx]

## Slide 1
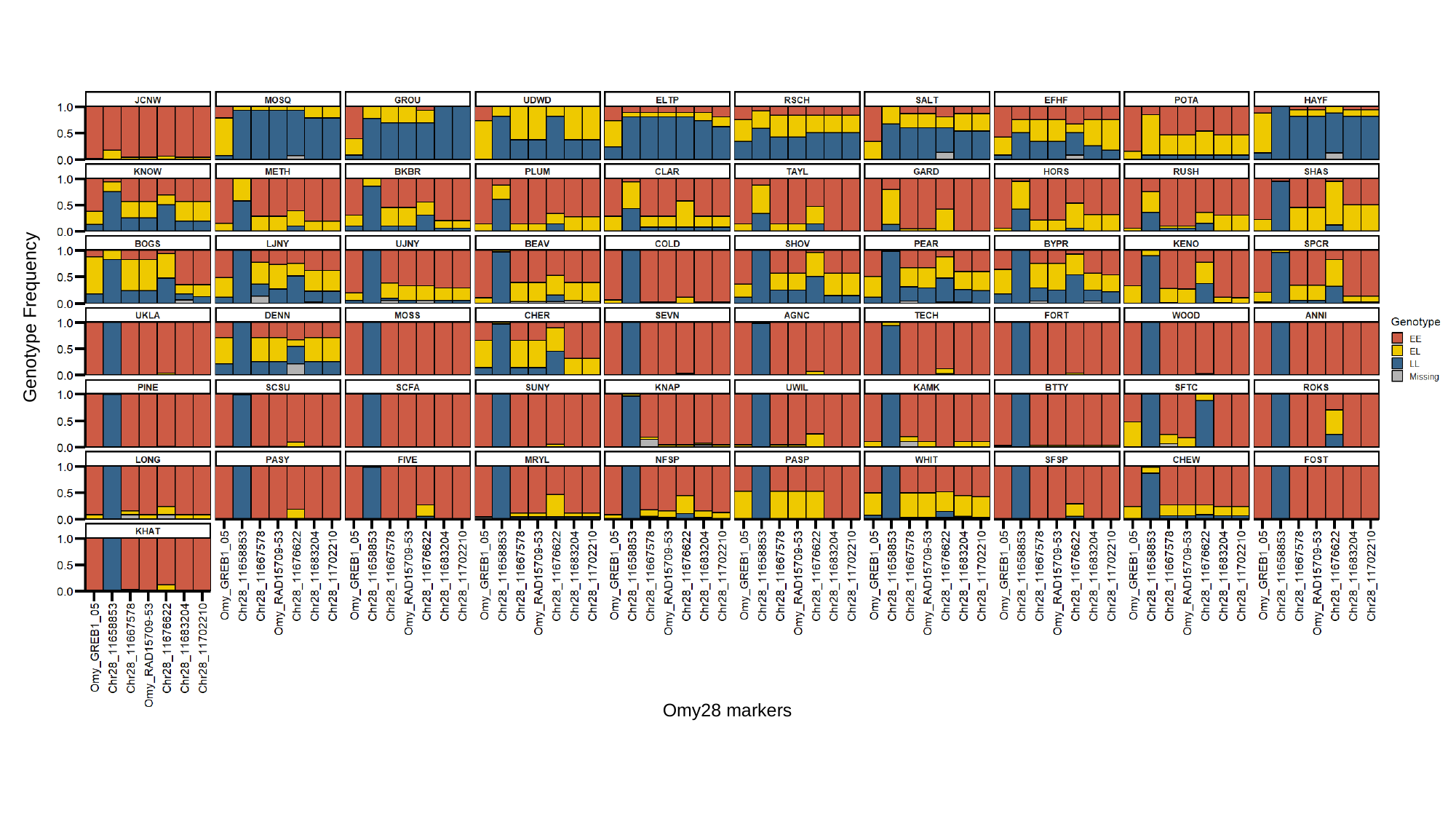

Genotype Frequency
Omy28 markers

Supplement: Supplementary file 6 — Figure S6: eva70297‐sup‐0006‐FigureS6.pptx. O. mykiss genotype frequencies across contemporary collections (n = 2081 individuals from 61 collections) at seven representative Omy28 markers associated with adult migration timing. Colors indicate early‐migration timing associated homozygous genotype (EE), heterozygous genotype (EL), late‐migration timing associated homozygous genotype (LL), and uncalled genotypes (Missing). [file EVA-19-e70297-s003.pptx]
