# Supplementary material for: Characterizing the Distribution of Oncorhynchus mykiss Genetic Diversity in the Klamath River Basin Before Dam Removal
Source: Evol Appl. 2026 Jul 12;19(7):e70297. doi: 10.1111/eva.70297 (PMC13358376; doi:10.1111/eva.70297)
Supplement: Supplementary file 7 — Figure S7: eva70297‐sup‐0007‐FigureS7.pptx. O. mykiss genotype frequencies across 13 locations with corresponding past and contemporary collections at representative Omy5 and Omy28 markers associated with anadromy/residency phenotypes and adult migration timing, respectively. In (a) past and (b) contemporary collections colors indicate anadromous associated homozygous genotype (AA), heterozygous genotype (AR), resident associated homozygous genotype (RR), and uncalled genotypes (Missing) at Omy5‐24370. In (c) past and (d) contemporary collections colors indicate early‐migration timing associated homozygous genotype (EE), heterozygous genotype (EL), late‐migration timing associated homozygous genotype (LL), and uncalled genotypes (Missing) at OmyRAD15709‐53. [file EVA-19-e70297-s001.pptx]

## Slide 1
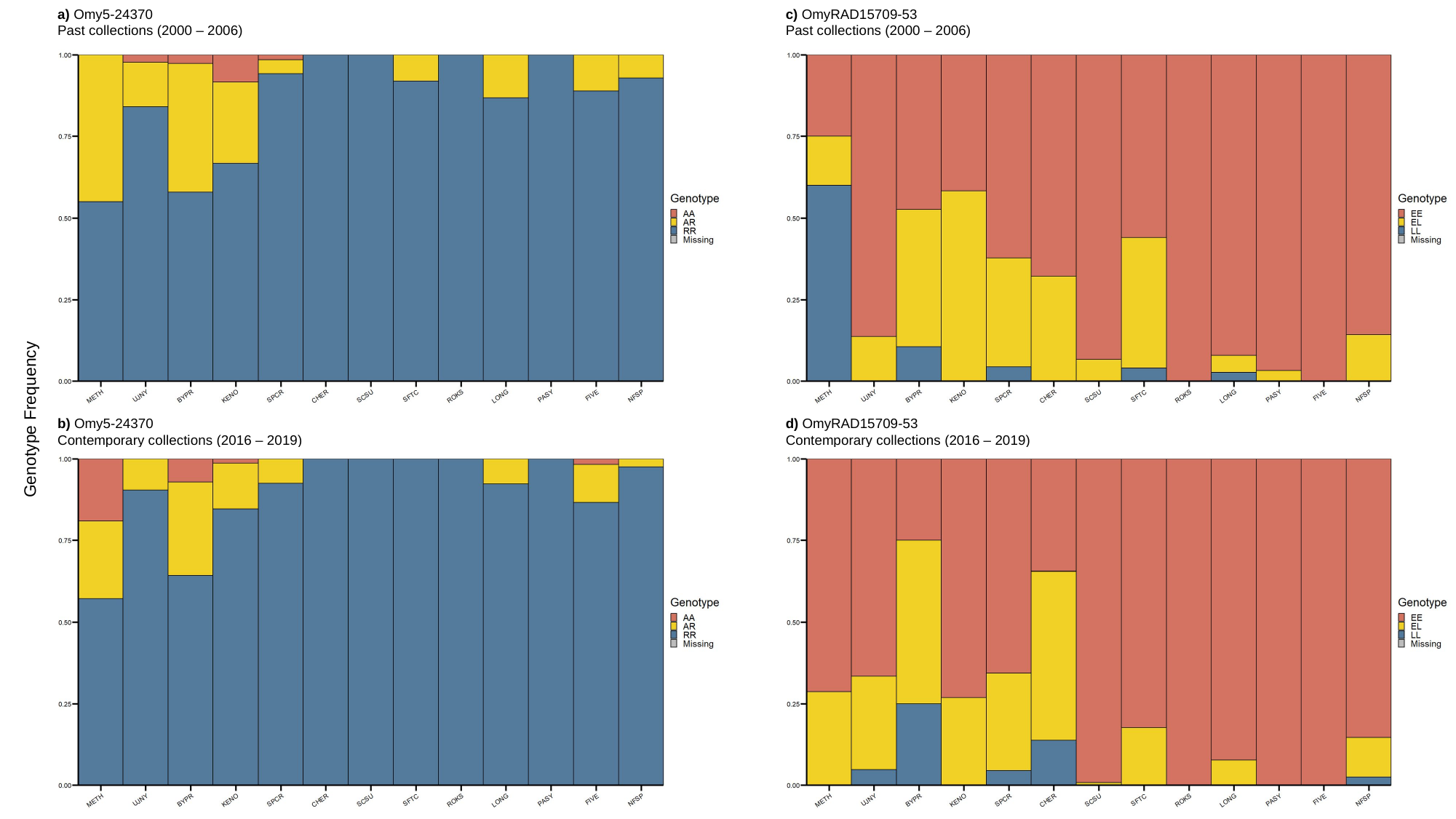

a) Omy5-24370
Past collections (2000 – 2006)
c) OmyRAD15709-53
Past collections (2000 – 2006)
Genotype Frequency
b) Omy5-24370
Contemporary collections (2016 – 2019)
d) OmyRAD15709-53
Contemporary collections (2016 – 2019)
