# Supplementary material for: Characterizing the Distribution of Oncorhynchus mykiss Genetic Diversity in the Klamath River Basin Before Dam Removal
Source: Evol Appl. 2026 Jul 12;19(7):e70297. doi: 10.1111/eva.70297 (PMC13358376; doi:10.1111/eva.70297)
Supplement: Supplementary file 10 — Table S3: Genotype to anadromy/residency phenotype associations of Omy5 markers characterized by Pearse et al. (2014). [file EVA-19-e70297-s002.docx]

**Supplementary Material for:** Characterizing the distribution of *Oncorhynchus mykiss* genetic diversity in the Klamath River Basin before dam removal

The anadromy/residency phenotypes of *O. mykiss* sampled in the Klamath River Basin were inferred based on genotypes at six Omy5 markers. To infer phenotypes, we compared genotypes in our dataset to those associated with anadromy/residency across watersheds in Southern Oregon and Northern California (Pearse et al. 2014).

Table S3. Genotype to anadromy/residency phenotype associations of Omy5 markers characterized by Pearse et al. (2014).

| **Omy5 Marker** | **Anadromy associated genotype** | **Residency associated genotype** |
| --- | --- | --- |
| OmyR19198Pearse | AA | GG |
| OmyR40252Pearse | AA | TT |
| OmyR14589Pearse | AA | TT |
| OmyR24370Pearse | AA | GG |
| OmyR33562Pearse | GG | AA |
| OmyR40319Pearse | TT | CC |

**References**

Pearse, D.E., Miller, M.R., Abadia-Cardoso, A., Garza, J.C. (2014). Rapid parallel evolution of standing variation in a single, complex, genomic region is associated with life history in steelhead/rainbow trout. *Proceedings of the Royal Society B*, 281, 20140012.
